# Microarray data can predict diurnal changes of starch content in the picoalga *Ostreococcus*

**DOI:** 10.1186/1752-0509-5-36

**Published:** 2011-02-26

**Authors:** Oksana Sorokina, Florence Corellou, David Dauvillée, Anatoly Sorokin, Igor Goryanin, Steven Ball, François-Yves Bouget, Andrew J Millar

**Affiliations:** 1 School of Biological Sciences, The University of Edinburgh King's Buildings, Mayfield Road, Edinburgh EH9 3JH, UK; 2 UPMC Univ Paris 06, UMR7621 LOMIC. Observatoire Océanologique, F-66651, Banyuls/mer, France; 3 CNRS, UMR7621 LOMIC. Observatoire Océanologique, F-66651, Banyuls/mer, France; 4 UGSF, UMR 8576 CNRS, USTL, Univ Lille Nord de France, F-59650 Villeneuve d'Ascq, France; 5 School of Informatics, Informatics Forum, The University of Edinburgh, 10 Crichton Street, Edinburgh, EH8 9AB, UK; 6 Centre for Systems Biology at Edinburgh, C.H. Waddington Building, King's Buildings, Mayfield Road, Edinburgh EH9 3JD, UK; 7 Institute of Cell Biophysics RAS, Pushchino, Moscow region, 142290, Russia

## Abstract

**Background:**

The storage of photosynthetic carbohydrate products such as starch is subject to complex regulation, effected at both transcriptional and post-translational levels. The relevant genes in plants show pronounced daily regulation. Their temporal RNA expression profiles, however, do not predict the dynamics of metabolite levels, due to the divergence of enzyme activity from the RNA profiles.

Unicellular phytoplankton retains the complexity of plant carbohydrate metabolism, and recent transcriptomic profiling suggests a major input of transcriptional regulation.

**Results:**

We used a quasi-steady-state, constraint-based modelling approach to infer the dynamics of starch content during the 12 h light/12 h dark cycle in the model alga *Ostreococcus tauri*. Measured RNA expression datasets from microarray analysis were integrated with a detailed stoichiometric reconstruction of starch metabolism in *O. tauri *in order to predict the optimal flux distribution and the dynamics of the starch content in the light/dark cycle. The predicted starch profile was validated by experimental data over the 24 h cycle. The main genetic regulatory targets within the pathway were predicted by *in silico *analysis.

**Conclusions:**

A single-reaction description of starch production is not able to account for the observed variability of diurnal activity profiles of starch-related enzymes. We developed a detailed reaction model of starch metabolism, which, to our knowledge, is the first attempt to describe this polysaccharide polymerization while preserving the mass balance relationships. Our model and method demonstrate the utility of a quasi-steady-state approach for inferring dynamic metabolic information in *O. tauri *directly from time-series gene expression data.

## Background

Multiple regulatory mechanisms ensure that physiological processes are synchronised with the environmental day/night cycle. Twenty-four hour biological rhythms are known to pervade the transcriptomes of the several organisms tested, for example, human, mice and higher plants [1-3] Diurnal fluctuations in metabolism should be taken into consideration in a number of applications ranging from the design of drug application protocols in medicine to the prediction of maximal biomass growth rate for plants and algae. Computational studies of the dynamics of metabolism could effectively facilitate these branches of research, giving predictions for the timing of the physiological phenomena of interest. At present, several ways to model and analyse metabolic systems have been established [4]. Detailed kinetic models make possible the analysis of all aspects of regulatory interactions in the metabolic system, but usually require lots of specific numerical information. Less complicated stoichiometry-based models utilize more general information for moiety- and mass-balance, so that analysis of the whole-organism metabolic network becomes possible, but such techniques allow the exploration of steady states only [5-7]. These models are useful in predicting maximum growth yields and viability of knockouts. When applied to a particular pathway, flux balance analysis (FBA) allows the prediction of pathway capability, and, by *in silico *deletions and overexpression analysis, examination of the potential regulatory steps and, hence, candidate drug targets [8,9].

Additional information available from transcriptome data can significantly improve the results obtained by FBA. A few successful approaches to incorporate the gene expression data into the flux model to infer the metabolic information have been made during the last decade. Among them is the method called E-flux has been proposed recently [10]. It operates directly with reaction constraints setting the maximum flux capacity in accordance with the gene expression level. This approach was successfully used in drug target predictions for *M. tuberculosis*. Although did not take post-transcriptional regulation into consideration, it was enough sufficient for analysis of the steady state reached by bacteria in response to environmental and genetic perturbations. The alternative Boolean-like tri-value mapping of gene expression states used to reveal tissue-specific enzyme activity, reported by Shlomi et al [11], which allowed post-transcriptional regulation to be accounted for, even in the absence of a biologically inspired objective function. Both approaches mentioned successfully inferred static metabolic information, while applications to the dynamics of the metabolic state are still limited, whereas we consider the dynamic regulation that is unavoidable in photosynthetic metabolism.

In higher plants diurnal expression patterns are evidently demonstrated by the genes encoding the central metabolic pathways including those involved in the carbohydrate storage metabolism [3,12]. Many plants accumulate a considerable amount of the CO_2 _fixed during the day in the form of starch, which is remobilised to sucrose during the night to avoid carbon depletion in plant cells [13]. Transcriptional regulation of starch metabolic genes by the circadian clock, by light/dark signals, and by sugars has been extensively characterised [13,14], but its functional importance is unclear. The measured activities of the cognate enzymes are arrhythmic or follow different temporal profiles [12], owing to a combination of post-translational and metabolic control mechanisms [15]. Among the latter, redox potential regulation appears the most significant for the events spatially related to the chloroplast, as it connects light, sugar and pH signalling in one highly organized scenario [16-18]. Transcriptome data has therefore been of little or no help in understanding the diurnal rhythms of starch metabolism in higher plants.

Eukaryotic green microalgae from the picophytoplankton (cells with a size <2 μm) shares the main photosynthetic functions with higher plants. However, the gene redundancy is reduced in these small genomes, thus making them attractive models for investigating the general properties of photosynthetic physiology. In addition to *Chlamydomonas reinhardtii *that has been used as a model organism for plant biology for the last half century, [19] recently, the genomes of four Prasinophyceae algae, *Ostreococcus *spp *tauri *and *lucimarinus *and two *Micromonas *species, have been sequenced [20,21].

**Figure 1:**
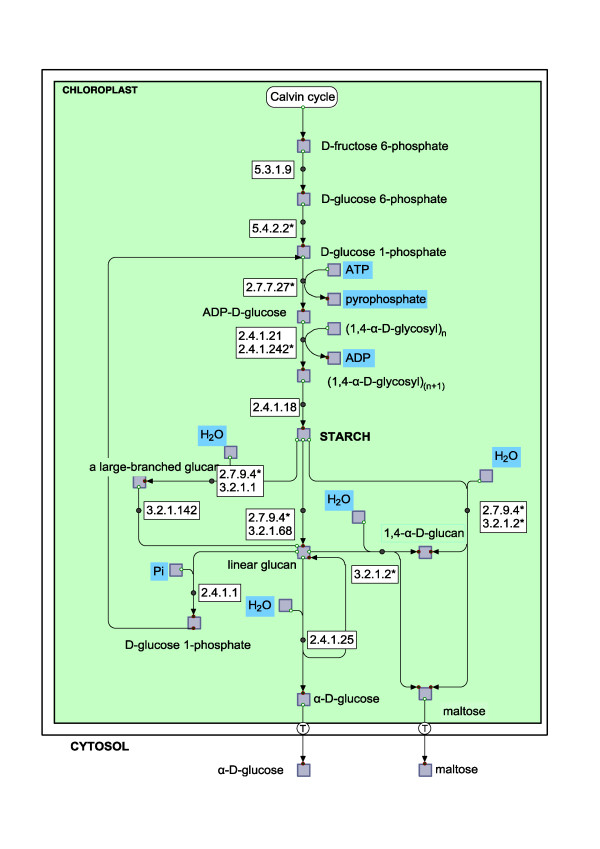
**The starch metabolic pathway in to the chloroplast**. EC numbers correspond to the respective enzymes: 5.3.1.9 - Glucose 6-phosphate isomerise (Ot11g02980); 5.4.2.2 - Phosphoglucomutase (Ot15g02630); 2.7.7.27 - ADP-glucose pyrophosphorylase (Ot07g03280, Ot07g02930, Ot20g00490); 2.4.1.21 - Starch synthase (Ot06g03410, Ot13g01250, Ot16g02790, Ot13g01230, Ot16g01560); 2.4.1.242- Granule bound starch synthase (GBSSI )( Ot06g03200); 2.4.1.18- Starch branching enzyme (Ot03g00840, Ot04g04110); 2.4.1.1 -Starch phosphorylase (Ot11g00280, Ot04g02110); 2.4.1.25- Disproportionating enzyme (Ot02g05750, Ot02g05750, Ot11g02300); 2.7.9.4- α-glucan, water dikinase (GWD) (Ot13g01510, Ot16g02370, Ot04g04170, Ot08g01260); 3.2.1.1 - α-amylase (Ot16g00380, Ot02g05490, Ot10g00260, Ot07g02010); 3.2.1.2 - β- amylase (Ot02g06980, Ot03g03190, Ot03g03170); 3.2.1.68- Isoamylase (Ot14g02550, Ot12g00310); 3.2.1.142- Pullulanase (Ot01g03030); MEX1- maltose transporter (Ot09g03160). Asterisks* point out the potential targets for the genetic regulation revealed by the simulations.

The small free-living phytoplanktonic eukaryote*, Ostreococcus tauri *[22,23] has an extremely simple cellular organization and very compact genome (12.5Mb) [20]. Despite the overall genome simplicity, with respect to polysaccharide metabolism, *Ostreococcus *demonstrates a structural complexity comparable to higher plants [24]. The ADP glucose-based starch metabolic pathway contains all the steps, characteristic of plants and *C. reinhardtii*, with multiple enzyme forms for each pathway step (Figure 1). The regulatory mechanisms for starch metabolism in *O. tauri *are likely distinct from those in plants. *O.tauri *accumulates starch within only one granule that takes up most of the space within a single chloroplast. The partitioning and division of the granule for sterical reasons should necessarily coincide with the division of the chloroplast and the cell itself [24]. At the same time, the redox state-mediated modulation of starch related enzyme activity has been reported as less essential for *C. reinhardtii *and *O. tauri*, at least at the level of ADP-glucose synthesis [25]. Results for transcript and protein abundance for the main rate-limiting step of the starch synthetic pathway ADP-glucose pyrophosphorylase (AGPase) in *C. reinhardtii *from Ral et al [26] suggest a greater importance of transcriptional regulation in this alga, compared with higher plants. Major input of transcriptional regulation as well as the orchestrated expression of genes related to chloroplast compartment over the light-dark cycle was recently reported [27].

Based on the above and taking into account that genetic processes are slower than metabolic ones, one can expect that if the metabolic fluxes in these organisms are sufficiently driven by transcriptional regulation, they could be directly approximated from gene expression data sets under diurnal conditions. Indeed, in the absence or weakness of posttranslational regulation and metabolic feedback, the starch metabolic subsystem could be approximated as comprised of two levels regarding the rate of the processes: slow processes leading and rapid processes following. The slow genetic level defines the state of the metabolic one by the specification of enzyme abundance. The rapid metabolic level reaches steady state much faster than the genetic level changes the concentration of enzymes. Thus, it could be treated as always being in the steady state, with the flux configuration specifically guided by gene productivity at a particular time point. In this paper we will show that such an approximation provides reasonable accuracy in predicting starch dynamics in *O. tauri*.

By accomplishing a full metabolic reconstruction of the *O. tauri *starch metabolic pathway, we developed a detailed reaction model that describes all the steps of starch biosynthesis and degradation known for the Chloroplastida. The model describes the process of polymerization with multiple approximated reactions for short, middle and long glucans to enable incorporation of all the gene essentiality. We obtained the gene expression (microarray) time series for *O. tauri *and integrated them to the flux model in a similar fashion as [10] in order to test the principle transcriptional regulatory pattern. This was impossible to do intuitively as gene family members showed very different experimental profiles, which could only be integrated in the detailed model. The method proposed allows the prediction of the flux distribution and temporal behaviour of key metabolites (starch and maltose) during the 12/12 light-dark cycle, which was successfully confirmed by empirical results. Single deletion analysis suggests the main regulatory steps responsible for the diurnal starch pattern and, thus, potential targets for genetic regulation.

## Results

We started from the exploration of the Kyoto Encyclopedia of Genes and Genomes databases (KEGG) to obtain the necessary information about the proteins that comprise the starch pathway. Information in KEGG is available for *O. lucimarinus *and is partially incomplete. Nevertheless, based on the results of the EST transcriptome analysis from [24] and BLAST sequence similarity search analysis, we were able to identify orthologous proteins for all the 16 enzymes and 2 transporters involved in the pathway, encoded by 43 genes (Table 1).

**Table 1:** *O. tauri *starch metabolism enzymes with corresponding genes

EC name	definition	gene name	Microarray available	ORF
4.1.2.13	Fructose-bisphosphate aldolase	fbaI	x	Ot01g03020
		fbaI	x	Ot03g00610
		fbaII	x	Ot10g01490
3.1.3.11	Fructose-1,6-bisphosphatase		x	Ot03g00330
			x	Ot14g01140
5.3.1.1	Triosephosphate isomerase		x	Ot09g00080
5.3.1.9	Glucose-6-phosphate isomerase		x	Ot11g02980
5.4.2.2	phosphoglucomutase		x	Ot15g02630
2.7.7.27	ADP-glucose pyrophosphorylase	AGPLU1		Ot07g03280
		agpsu1	x	Ot07g02930
		agplu2		Ot20g00490
2.4.1.21	Soluble Starch synthase	SSIII-C		Ot06g03410
		SSIII-B	x	Ot13g01250
		SSII	x	Ot16g02790
		SSI	x	Ot13g01230
		SSIII-A	x	Ot16g01560
2.4.1.242	Granul-bound starch synthase	gbssI	x	Ot06g03200
2.4.1.18	Starch branching enzyme	SBEII(?)	x	Ot03g00840
		SBEI	x	Ot04g04110
2.4.1.1	starch phosphorylase			Ot11g00280
			x	Ot04g02110
		SPho1		Ot11g01020
2.4.1.25	disproportionating enzyme	DPE1	x	Ot02g05750
				Ot11g02290
				Ot11g02300
3.2.1.1	α-amylase	Aamy1	x	Ot16g00380
			x	Ot02g05490
		Aamy2	x	Ot10g00260
		Aamy3	x	Ot07g02010
3.2.1.2	β-amylase		x	Ot02g06980
		Bamy2	x	Ot03g03190
		bamy1		Ot03g03170
3.2.1.68	Isoamylase (debranching enzyme)	dbe1 (Isa1)	x	Ot14g02550
		Isa2		Ot02g07230
		dbeII (Isa3)	x	Ot12g00310
3.2.1.142	Pullulanase	spu	x	Ot01g03030
2.7.9.4	α-glucan, water dikinase (GWD)	SR1-A	x	Ot13g01510
		R1 C		Ot16g02370
				Ot04g04170
	Glucose transporter			Ot03g05590
	Glucose transporter			Ot14g01870
	Glucose transporter			Ot08g01260
	Maltose transporter	MEX1	x	Ot09g03160

### Transcripts encoding starch metabolizing enzymes in O. tauri show distinctive diurnal patterns

We used microarrays to obtain the expression profiles of genes encoding enzymes of the starch metabolic pathway. For starch synthesis, we included genes annotated as encoding chloroplastic phosphoglucoisomerase (PGI) and phosphoglucomutase (PGM), a small subunit of ADP-glucose pyrophosphorylase (AGPase), starch synthase (SS, 5 isoforms, including GBSSI), and starch branching enzyme (SBE, 2 isoforms). For the starch degradation pathway, we include glucan, water dikinase (GWD), α-amylase (AMY, 4 isoforms), β-amylase (BAMY, 2 isoforms), isoamylase (ISA1 and ISA3 isoforms), pullulanase, starch phosphorylase, glucose and maltose (MEX1) transporters. In total we incorporated expression profiles for 29 out of the 43 genes annotated as starch-related into the analysis, which uniformly cover the whole pathway.

**Figure 2:**
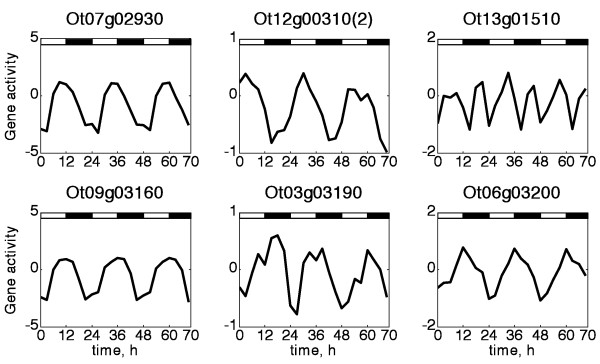
**Diurnal mRNA expression profiles for selected genes**. From left to right, upper row: ADP-glucose pyrophosphorylase, (Ot07g02930), isoamylase ISA3 (Ot12g0310), GWD (SEX1) (Ot13g01510). Lower row: maltose transporter MEX1 (Ot03g03160), β-amylase (Ot03g03190), GBSSI (Ot06g03200). The remaining profiles are presented in Additional file 1, Figure S6.

Single genes for PGI and PGM show very similar profiles, reaching their maximum level in the first 3 hours of light, and decreasing throughout the next 12 hours with a minimum during the early night (Additional file 1, Figure S6). The RNA for the small subunit of the AGPase attains its minimum level in the first 3 hours of light, increasing during the following 6 hours and having a peak just before the light-dark transition (Figure 2).

Transcripts for 3 starch synthase isoforms (Ot16g01560, Ot13g01230, Ot13g01250), annotated as SSI and SSIII- A and B (Table 1) change in a coordinated way over LD cycle, decreasing during the day and increasing during the night (Additional file 1, Figure S6). Interestingly, all three isoform expression profiles demonstrate double peaks in the late night and early morning. A similar pattern could be observed in expression profile of one of isoamylase isoforms, ISA1 (Ot14g02250), which might indicate its participation in starch synthesis, as it has been reported previously for *Chlamydomonas *and *Arabidopsis *[28,29]. In contrast, the RNA abundance for isoamylase isoform ISA3 (Ot12g00310) decreases during the light period and increases during the night, thus, supporting the idea of its preferable functionality as a starch degrading enzyme [29].

RNA for SSII (Ot16g0790) has a different profile, staying relatively stable for most of the time with rapid changes connected to the light-dark transitions. Granule-bound starch synthase GBSSI (Ot06g03200) demonstrate the shape similar to the small AGPase subunit (Figure 2). One of SBE isoforms, SBEII (Ot03g00840) closely repeats the expression profile for SSI and III, while another one, SBEI (Ot04g04110), is 6 hours delayed with a maximum expression in the middle of the subjective day and a minimum at the end of the night (Additional file 1, Figure S6).

Transcripts encoding enzymes for starch breakdown demonstrate variability in expression profiles; nevertheless, several genes behave very similarly. Among them are two α-amylase isoforms (Ot07g02010, Ot10g00260), β-amylase (Ot02g06960), pullulanase (Ot01g03030), starch phosphorylase (Ot04g02110), MEX1 (Ot09g03160) and SEX4 (Ot13g02940). All of them peak at the subjective dusk and decrease during the night achieving the minimum level before dawn (Figure 2 and Additional file 1, Figure S6). Profiles for two α-amylase isoforms (Ot16g00380 and Ot02g5490) demonstrate a similar shape. The only isoform of GWD available in the experiment exhibits rhythmic expression with a 12 hour period, peaking in the middle of the day and night periods (Figure 2).

Although the peak expression of this set of starch biosynthetic genes occurs in the day time and expression of some genes corresponding to starch degrading enzymes peaks during the night, we do not observe a strict relationship between the time of peak expression and the proposed function. For instance, several β-amylases and α-amylases are expressed during the day, whereas some starch synthases and the AGPase small subunit are equally or preferentially expressed during the night (Figure 2 and Additional file 1, Figure S6). This variability prevented a simple prediction of the starch accumulation profile, so it was attractive to integrate the combined effect of all gene expression profiles on the diurnal starch pattern, using a mathematical model.

### Model construction

To make the model's constraints more specific we included in the model the chloroplast-located gluconeogenic subsystem, in order to embrace all the events for polysaccharide storage from the point of release of glyceraldehyde-3-phosphate (GAP) from the Calvin cycle, so that we used 16 enzymes and 43 genes in total (Figure 1 and Additional file 2, Figure S1). The model represents the approximated direct pathway for carbon starting from CO_2 _fixed in photosynthesis, through starch granule formation, to glucose and maltose obtained from starch degradation. Restricted in such a way, the model is well bounded in terms of subcellular organisation as well as a defined mass exchange with the rest of the cell. As we constrained ourselves to one compartment, the model includes only those reactions that are considered chloroplast specific, and transport to and from the cytosol (in a form of exchange reactions). Only those genes that were annotated as chloroplast-located were connected to the corresponding fluxes. The process of starch degradation is described up to the departure of glucose and maltose to the cytosol (Additional file 2, Figure S1, Additional file 3, Table S1).

The model of the starch metabolic pathway comprises all the steps known for the higher plants and green algae [30,31] (Figure 1). It starts with the conversion of glucose 1-phosphate (G1P) to ADP-glucose by means of ADP-glucose pyrophosphorylase (2.7.7 27)[32]. ADP-glucose is used as a building block for the extension of linear glucan chains by both the soluble starch synthase (2.4.1.21) and granule-bound starch synthase (2.4.1.242)[26,31,33,34]. The starch branching enzyme (2.4.1.18) acts on the linear glucans producing branched glucans [35]. The defined ratio of long linear and highly branched glucans gives the starch granule content.

Starch degradation is initiated by starch granule phosphorylation carried out by alpha-glucan, water dikinases (GWD, 2.7.9.4)[17]. Phosphorylated starch molecules can be debranched by the starch debranching enzyme (ISO, 3.2.1.68) and pullulanase (3.2.1.242), and hydrolyzed by α-amylase (3.2.1.1) giving short linear glucans, and by β-amylase (3.2.1.2), producing maltose and maltotriose [33]. The latter could be disproportionated to glucose and short glucan by means of the disproportionating enzyme (DPE1, 2.4.1.25)[33]. Long linear glucans are also subject to the action of starch phosphorylase (2.4.1.1) giving G1P and short linear glucans. Maltose, the main product of starch degradation, and glucose, are considered as the only products of starch mobilization that leave the chloroplast; their release occurs through specific transporters [36,37].

Although the overall starch metabolism constitutes a multistep polymerisation process, it is still possible to simplify the complexity by reducing the number of metabolites to a presentable minimum, enabling one to illustrate the most essential biochemical features (Additional file 2, Figure S1). To our knowledge this is the first detailed model of this kind for the polysaccharide storage metabolism.

The model of starch metabolism contains 37 metabolites and 69 reactions (Additional file 3, Table S1), mediated through 17 enzymes (42 genes). The key model assumptions are listed below:

- Gene expression level could be used as a sufficient estimator of relative activity of the reaction in the pathway.

- Starch consists of amylose and amylopectin in the approximate ratio 1:4 [30]. Our model starch consists of long linear glucans (100 glucose residues) and large branched glucans (100 and 120 glucose residues), thus we consider starch as a composite of three main components: 100LG (long linear glucan of 100 glucose residues), 100BG (branched glucan of 100 glucose residues) and 120 BG (branched glucan of 120 glucose residues) in the ratio 1:2:4. The exchange reaction for starch formation in our model, thus, has the form of: **100LG-g + 4 100BG-g + 2 120BG-g **<=>, that fixes the ratio.

The rest of the model assumptions can be found in Additional file 4 (Part 1).

### In silico investigation of each gene's relative impact on the overall starch production

We started by investigating each gene's relative impact on the overall production of starch. In other words, we aimed to track out how the proposed profile of starch flux over the diurnal cycle could be affected by the rhythmical expression of each particular gene, and identify those genes that provided the maximum contribution. For this we applied each gene's microarray data, one by one, modifying the upper bound for each model reaction flux in compliance with the corresponding microarray value (see Materials and Methods for the details). Introducing a certain gene expression profile, we simulated the situation in which all the genes, except the chosen one, were expressed at their maximum level. Thus, we interrogated how the flux limitation obtained from single gene regulation influenced the overall starch production flux (Additional file 1, Figure S7).

We found that β-amylase (Ot03g03190) and AGPase (Ot07g02930) strongly affect the 'light' part of the starch flux pattern, while GWD (Ot03g01510), both ISA1 and ISA3 (Ot12g00310 and Ot14g02550), and α-amylase (Ot07g02010) have an influence upon the 'dark' region (Additional file 1, Figure S7, B and C). While for the AGPase the result is quite predictable, as it corresponds to the main rate-limiting step in the starch biosynthetic pathway, for β-amylase its appearance as a key contributor for the starch production is less obvious. This could be explained from the model structure, as malto-oligosaccharides (MOS) are allowed to contribute to amylopectin synthesis (Additional file 3, Table S1 and Additional file 2, Figure S1). In the model maltotriose, one of the main products of β-amylase-mediated hydrolysis, is utilized by the D-enzyme and, together with the obtained longer-chain glucans (20LG and 40LG), may participate in starch synthesis. That might be the reason for β-amylase to stand out with respect to the starch synthetic flux in this simulation. The similar effects of both isoamylase profiles are also surprising as they are expected to be functionally opposite. Nevertheless, in the simulation they both change the shape of the "dark" region towards synthesis (Additional file 1, Figure S7, B and C). Results for glucan, water dikinases and α-amylases affecting "dark" region of the starch pattern are accordant with their proposed functionality.

The above observations do not provide a completely accurate description of starch flux during the light/dark cycle as the analysis of flux limitation for each particular gene gives quite a patchy representation. Since in the metabolic pathway the products of different genes are usually working in concert, we moved to analysis of the combined action of genes with different expression profiles.

### In silico investigation of the combined influence of all the genes on the starch diurnal pattern

**Figure 3:**
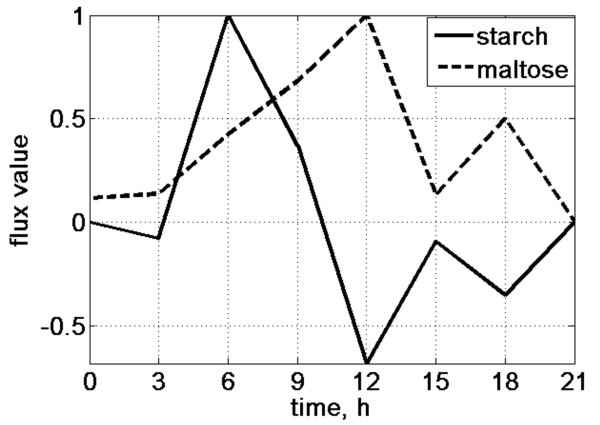
**Simulated diurnal rhythms of starch and maltose production fluxes**. All available gene expression data over the 12 h light:12 h dark cycle is included. The peak in maltose flux at time 12 evidently corresponds to the trough of the starch flux.

We interrogated the combined impact of all the genes involved in the pathway, substituting the entire given microarray data *simultaneously *to track the fluxes for starch and maltose production during the day. For this, at each time point all the reactions' upper bounds were modified by the same method as above. As before, we introduced light:dark cycle (LD) by allowing the uptake of CO_2 _from time 0 until time 12 (Figure 3). For comparison, if all the upper bounds are set to their maximum value (unit in our case), we obtain the simple On-Off step function shape for diurnal starch flux, which corresponds to our knowledge about the purely autotrophic nature of *O. tauri *(Additional file 1, Figure S8). We also simulated the cases where only the reaction upper bounds of genes involved in gluconeogenesis are modified. Additional file 1, Figure S8 shows that those reactions have an influence on a resulting starch flux at the time points 6 and 9 h.

We obtained the maximum starch synthesis flux at time 6 and the peak degradation flux at time 12. Interestingly, we observed a coinciding peak of maltose export flux at time 12, which is biologically meaningful, as the maximum rate of maltose production must be related to the maximum rate of starch degradation. Figure 3 illustrates the negative correlation between starch and maltose fluxes. The time point time 12 corresponds to the light-dark transition.

It is known for plants that the biggest release of maltose takes place in the middle of the night [36]. Nevertheless, as far as the chloroplast division and the attendant division of the starch granule in *Ostreococcus *coincides with cell division and happens at the end of the day [24], it is not inconceivable that these events might dictate the timing for the maximal starch elongation and degradation.

Additional file 1, Figure S9 shows the flux distribution obtained within each time point. At time zero there is no starch production, and CO_2 _is already available. The system converts the remaining short glucans to maltose and glucose. At time point time 3 we observe quite an unexpected situation. Regardless of the CO_2 _uptake and ADP-glucose synthesis, the system operates towards starch degradation. This reflects the combined action of gene expression data and should be clarified with experiments, but to speculate, it may be accounted for by an increased need for a rapid sugar supply in the early day. At time 6 we see the normal starch assimilation pattern with all steps of glucan synthesis participating. The same is correct for time 9. However, it should be noted that the absolute flux value is smaller for the reactions involved in starch granule formation (100LG_s, 100LG_g, etc.).

At time 12 the starch flux leaves the positive region, and the process of degradation starts to dominate. The fluxes of the reactions for starch granule phosphorylation are brought to the forefront; meanwhile, the flux for maltose export attains its maximum value (Additional file 1, Figure S10).

At time 15 and time 18 starch degradation is proceeding, though the fluxes have relatively smaller values. At time 21 all the fluxes are equal to zero (not shown). This is caused by a zero value for maltose export flux, which, in turn, results from a zero value for the expression level of MEX1. In our procedure, this value is directly applied as the upper flux bound for the reaction of maltose translocation from the chloroplast. However, the real flux distribution at the time point time 21 merits further experimental investigation.

### In silico investigations of the dynamics of starch content confirmed by experiments

**Figure 4:**
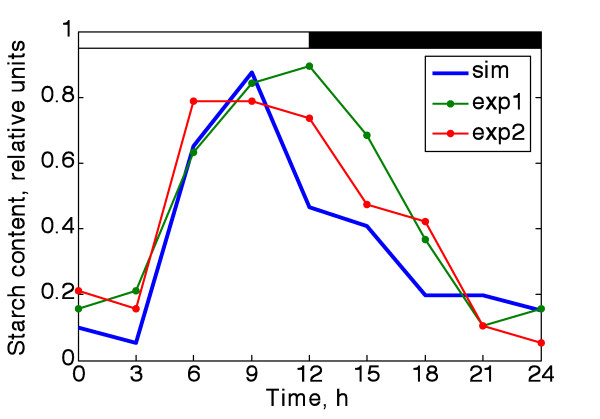
**Diurnal pattern of starch content obtained by simulation and experiment**. The diurnal pattern of starch content obtained from two independent experiments is shown in comparison with the simulated starch profile, derived as a cumulated flux value. The details of the starch assay are presented in Materials and Methods.

To obtain the diurnal dynamics of the starch content from the flux, we estimated the cumulate of the starch flux at each time point. We also measured the actual starch content dynamics during the 12/12 light-dark cycle experimentally (see the experimental protocol in the Materials and Methods). Figure 4 shows the comparison of the predicted pattern for the starch content with that obtained from two independent experimental replications. The predicted curve matches the real starch content behaviour with a sufficient level of accuracy. Note that the decrease in starch content during the first three hours in one of the replicates precisely follows the simulation. Also the shape of the simulated "light" region stays very close to the experimental one until the time of the light-dark transition. The maximum of the starch content in the model falls after about 10 hours of light, whereas the measured decrease in starch content appears to be delayed by about 2-3 hours. This may occur because the current model lacks the regulatory events that are likely to accompany the light-dark transition, so the only deficiency of the model is located around the time of dusk. Nevertheless the overall predicted starch profile is in good agreement with the reality and the steady regions of the cycle are described quite well. Another possible explanation is cell division, which happens close to the light-dark transition [38], with a slightly different rate in each experiment and strongly depends on the culture density.

While simulating the longer-term behaviour of starch content, we observed the tendency for its baseline to increase (Additional file 1, Figure S11). The overall starch production/degradation ratio is clearly displaced towards production, giving a positive overall starch flux equal to 0.4. Given the necessity for cell volume increase and starch granule division, we can conclude that the trend toward net starch accumulation is realistic enough. One consequence is that starch never degrades completely. This result is indirectly supported by the fact that neither for *Ostreococcus *nor for *Chlamydomonas *has full starch deprivation been demonstrated, even after prolonged incubation in darkness [20,23].

However, it must be stressed that all the model predictions were calculated for a single cell, so the estimates of the positive trends for the starch flux and content may arise from the fact that no dilution coefficient term was included in the model.

### Single gene deletion in silico experiment

**Figure 5:**
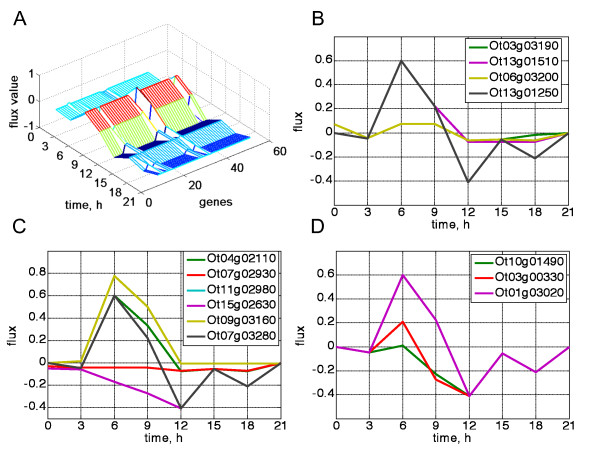
***In silico *single gene deletion experiment**. A. A 3D representation of the results of a single gene deletion experiment. B. Effects of deletion of GBSSI (Ot06g03200) (GBSSI), GWD (Ot13g01510) or β-amylase (Ot03g03190) on the overall starch flux diurnal pattern. SSIII-B (Ot13g01250) corresponds to the unperturbed starch pattern. C. Effects of deletion of AGPase (small subunit, Ot7g02930), phosphoglucomutase or glucose 6-phosphate isomerise (Ot15g02630 and Ot11g02980, traces overlay each other), MEX1 (Ot09g03160), starch phosphorylase (Ot04g02110) on the overall starch flux diurnal pattern. AGPase, large subunit (Ot07g03280) corresponds to the unperturbed starch pattern. D. Effects of the deletion of fructose-1,6-bisphosphatase, fbaII (Ot03g00330) or fructose-bisphosphate aldolase (Ot10g01490) on the overall starch flux diurnal pattern. Fructose-1,6-bisphosphatase, fbaI (Ot01g03020) corresponds to the unperturbed starch pattern.

We aimed to identify the genes whose deletion affects the periodic behaviour of starch accumulation. In our *in silico *experiment, all the genes, except for the one selected for deletion, are rhythmically expressed following their respective microarray profiles. Each gene's expression value was set in turn to 0.01 to mimic gene knock out (or marginal state of knock down). After this, the simultaneous effect of the substitution of all genes was calculated as in the previous simulation (Figure 5). The 3D representation in Figure 5, A, shows three potential regions of interest that could be further analyzed. From the first 8 genes, the pattern is affected most impressively by deletion of the GBSSI (Ot06g03200) gene. This is quite a predictable result as the enzyme is responsible for the elongation of long linear glucans (Figure 5, B). Also, the 'dark' region of the pattern is sensitive to the deletion of GWD (Ot13g01510) and β-amylase (Ot03g03190); both straighten the pattern after time 12, decreasing the degradation rate.

From the middle 7 genes the most striking effect is induced by the deletion of AGPase (Ot7g02930) (AGPase), which removes the oscillatory pattern completely when deleted (Figure 5, C). This is again the main rate-limiting step of the biosynthetic pathway and is a potentially very sensitive regulatory target. The phosphoglucomutase (Ot15g02630) deletion moves the starch flux to the negative region, towards degradation. This enzyme controls the region of the linear part of the model landscape and is encoded by a single gene. Hence, the complete deletion of any step of the linear path will expectedly affect the productivity of starch synthesis.

The deletion of the maltose transporter MEX1 (Ot09g03160) increases the rate of starch production as it decreases the maltose efflux. Interestingly, starch phosphorylase (Ot04g02110) gene deletion changes the local pattern for the light-dark transition causing a noticeable decrease in the starch degradation rate during the dark period.

From the last 5 genes we can observe the deletion of fructose-1,6-bisphosphatase (Ot03g00330) gene, decreasing the starch synthetic flux (Figure 5, D). As in the case of phosphoglucomutase, this stems from the model structure where 1, 6 bisphosphatase in the initial linear region of the pathway determines the rate of the glucose entrance for starch synthesis. In a similar way, fructose-bisphosphate aldolase (Ot10g01490) deletion blocks starch synthesis during the day as it prevents the input of CO_2 _to the pathway.

For the maltose flux the results of the simulation of single gene deletion are presented in Additional file 1, Figure S12. Here, the deletion of β-amylase (Ot03g03190) abolishes the second maltose peak that happens at time 18 (Additional file 1, Figure S12, B). As β-amylase is the main maltose supplier in the system, this effect is quite obvious. Both the deletions of the GBSSI (Ot06g03200)) (Additional file 1, Figure S12, C) and AGPase (Ot07g02930) (Additional file 1, Figure S12, D) genes remove the maltose pattern completely. These enzymes are among the key players in our model starch production pathway, and their disappearance leads to a significant decrease in the amount of the maltose precursor. Nevertheless, a nonzero maltose level remains due to maltose production from short glucans. Meanwhile, the deletion of the maltose transporter (MEX1) switches the maltose export off, as it blocks maltose flux completely.

Glucan, water dikinase (Ot13g01510) defines the kinetics of 'dark' maltose region (Additional file 1, Figure S12, C). This enzyme accounts for the starch granule phosphorylation that precedes starch degradation. This is why the deletion of the corresponding gene prevents the normal sequence of the events related to starch degradation during the night. Starch phosphorylase together with β-amylase constitute the two main sinks of the model system; the availability of the starch phosphorylase gene (Ot0402110) affects the magnitude of the first maltose peak and β-amylase affects the existence of the second one (Additional file 1, Figure S12, D).

## Discussion and Conclusions

In this paper we present novel method for predicting the timing of physiological processes based on time series gene expression data. We have chosen the starch pathway as it corresponds to a key metabolic process, which has an impact on the whole organisms vital functions during the photoperiod. We also see a strong variability within the some isozyme families. At the same time, it represents a particular subsystem, which is finely biologically bounded with distinct input and outputs that could be directly measured. Our experimental results for diurnal RNA profiling do not demonstrate the expected homogeneity in the distribution of timing patterns and, thus, do not allow an intuitive classification of the "day" and "night" enzymes with respect to their proposed function. Namely, some important starch synthetic proteins show peak expression at the onset of the dark, while transcripts for a few starch degrading enzymes peak during the early light period. The combined action of all the genes is of great interest and could be interrogated only within a detailed pathway model.

We developed a detailed reaction model of starch metabolism, which, to our knowledge, is the first attempt to describe the polysaccharide polymerization while preserving the mass balance relationships (generics like (1,4-glucan)_*n *+ 1 _that are common representation for this pathway do not allow mass -balance modelling). Previous reaction models for carbohydrate metabolism usually present starch synthesis as ADP-glucose being converted to starch without intermediate stages [39]. Although this is a useful simplification when the whole organism metabolism is modelled, it does not allow the investigation of the full complement of genes involved.

We analysed the dynamics of starch during the light/dark cycle as a sequence of metabolic quasi-steady states, each of them corresponding to particular genetic state within the 3 hour interval. This approximation correctly predicted the diurnal pattern of starch content in *O. tauri *in our experimental observations.

Obviously, neither mRNA nor even protein abundance itself may be sufficient for prediction of true flux through a reaction, but it could be the effective indicator of the relative strength of the flux over a time course. At the same time, modification of the upper bounds of reaction fluxes is an effective way to model genetic knockout experiments using FBA and combining FBA with boolean gene models.

The model structure and the constraints applied are sensible and biologically plausible for they are based on the actual pathway structure and describe the main features of starch metabolism. The model starch "phenotype" on the whole matches the data from [24], taking starch partitioning and degradation to the nightfall. We predicted the increase in maltose outflow starting from time 3 with a peak at time 12. The revealed timing seems physiologically reasonable, as the cell should accumulate a sufficiently large polysaccharide store up to the moment of its distribution between the two daughter cells, which occurs during the light-dark transition [24].

We identified the set of potential targets of transcriptional regulation (Figure 1 and Additional file 3, Table S2). The main player from the list is AGPase, which has already been reported as catalysing a key regulatory step for *Arabidopsis *and *Chlamydomonas*. The list also includes GBSSI, β-amylases, glucan, water dikinases GWD (R1) and the maltose transporter MEX1. As we observed, the main regulation targets are concentrated at the linear entrance to the system, within the actual topological bottleneck of the system (Fructose-1,6-bisphosphotase, AGPase, etc). Some of the events of high importance, corresponding to synthesis and degradation, are located at the granule surface. Here GBSSI and GWD appear as the main potential targets for genetic regulation. The final set of targets deals with the production of maltose and its withdrawal from the system. The main target enzymes identified for this location are β-amylases and the maltose transporter MEX1.

The results of our robustness analysis are generally in agreement with the results of gene perturbations, as the genes implicated successfully match the 'sensitive' reactions. For instance, the reaction of 20LG glucan synthesis is carried out by the GBSSI enzyme that appears in all the lists. The presence of debranching reactions like '100BGs_Pto20LG' and '60BGto20LG' is accompanied by genes for the debranching enzyme. However, although the genes for glucan, water dikinase perform as attractive regulatory targets, the starch flux is not very sensitive to small fluctuations in fluxes of the corresponding reactions until they attain some significant threshold (Additional file 1, Figure S4). Hence, for presumptive conclusions about the nature of regulation, both the genetic and also the biochemical and metabolomic data have to be taken into account.

### Comparison to previous approaches

A number of approaches for integrating static gene expression data in constraint-based modelling were published recently [10,11]. Shlomi et al., for instance, proposed a method to take into account tissue specificity in metabolic models by incorporating tissue-specific gene expression states into the flux optimisation scheme [11]. This method determines the FBA solution together with maximising the correspondence between expression status and metabolic fluxes. However, for our purposes the representation of expression levels with their discrete, tri-valued method seems an oversimplification and leads to the destruction of any dynamical pattern, especially when more than one isozyme is responsible for reaction regulation (data not shown). For this reason we incorporated the time-dependant activity of gene expression into our flux model directly, modulating the upper bound of each flux.

Such modification of flux upper bounds is a common method in the prediction of knockout and knockdown effects in metabolic models. The main difference here is that we do not completely switch any flux off but gradually change its maximal value. Hence, here, for the first time, we have applied an expression time series to the analysis of the dynamics of a metabolic system. Our method resembles the E-flux method where the flux upper bound constraints are also represented as a function of measured gene expression, though this was not applied to dynamic data [10]. In place of the normalisation within a time point in E-flux, we have normalised the expression profile of individual gene over the whole time series. This approach will bring a growing volume of time series data to bear in studying metabolism.

### Transcriptional regulation is significant in unicellular algae

The approach presented here allows the derivation of diurnal metabolic information directly from the gene expression data. This implies some kind of linear correlation between the gene expression level and metabolic flux, which is evidently an over-simplification but similar concepts have been applied successfully [10]. The presence of post-transcriptional modifications and metabolic feedback will influence the results obtained by this method, not only because of the alteration of the steady state *per se*.

Figure 4, however, illustrates the high agreement between the model predictions and the actual dynamics of starch content, which suggests that this assumption was reasonable, at least for *O. tauri*. The reasons for such a strong influence of genetic regulation in unicellular algae are currently a matter of speculation. One possible explanation is the moderate level of post-translational regulation, in particular the lower level of redox regulation of starch metabolism as compared to the higher plants.

In photosynthetic organisms a wide range of chloroplast metabolic pathways have been shown to be redox regulated, especially those located to the chloroplast [40]. The major influence is exerted by light; nevertheless, sugars, namely sucrose and glucose, are also involved in redox signalling [40,41]. Hence, information from both metabolism and the environment is integrated through redox regulation to provide the appropriate growth and developmental responses.

The photosynthetic unicellular red algae *Galdieria sulphuraria *has been studied in respect of the redox regulation properties of several enzymes involved in the Calvin cycle [42]. Intriguingly, despite the high sequence similarity of the *G. sulphuraria *enzymes to those of other photosynthetic organisms, poor or no redox regulation of Calvin cycle has been found [42]. It appears that the level of the redox regulation itself could be a matter of adaptation, and for the land plants with their highly organized tissue and organ specificity, rapid adjustments to physiological and environmental changes could be of more importance than for marine unicellular algae.

**Figure 6:**
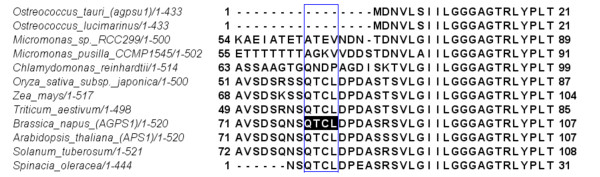
**Multiple alignments for the small (beta) subunit of ADP-glucose pyrophosphorylase**. Presented alignment includes sequences from *Arabidopsis thaliana*, *Solanum tuberosum*, pea, wheat, maize, *Ostreococcus tauri *and *lucimarinus*, *Chlamydomonas reinhardtii *and *Micromonas pusilla *and *CCMP490*. The sequence QTCL characteristic for the higher plants is absent in the unicellular algae.

Based on existing data for *Galdieria sulphuraria *and *Chlamydomonas reinhardtii*, we considered whether the level of redox regulation of the starch metabolic pathway in *Ostreococcus *also differs from higher plants. To explore this hypothesis more narrowly, based on literature and sequence databases (Uniprot, JGI), we checked whether enzymes of the starch metabolic pathway in *O. tauri *contain the target sequences reported as responsible for the redox-related modifications for plant species, namely, for AGPase, GWD and β-amylase enzymes [16-18]. Our sequence analysis revealed that in addition to already published results for *O. tauri *[25] and *C. reinhardtii *[24], neither AGPases of *O. lucimarinus *nor both *Micromonas *species contain the redox-target sequences (Figure 6). The same is true for another known redox regulated enzyme, GWD, which is believed to initiate the starch mobilization process. Also, we did not find the analogue for the redox regulated plant β-amylase isoform TR-BAMY (Additional file 4, Part 3). Therefore, it is possible that due to the relative weakness of the redox regulation of the starch pathway the genetic regulation to some extent takes up this role in algae. Interestingly, the same steps that are reported as redox regulated in plants appear to be the main targets of genetic regulation in our analysis, namely AGPase, GWD, β-amylase and MEX1 (Figure 1).

Allosteric regulation is also important in the control of starch metabolism in higher plants, but here again, according to [25], the regulation of *O. tauri *AGPase differs remarkably from plant enzymes. On the one hand, the small subunit appears to stay in a slightly preactivated state irrespective of the presence of the activator (3-PGA) and demonstrates low sensitivity to inhibitor (PPi). On the other hand, the large subunit, being fully catalytically competent, is assumed to be the only one that undergoes the allosteric regulation at subsaturated concentrations of effectors [25]. As our modelling and experiments used saturating light during the "day" period, the light-dark transitions appear to be the only intervals when this allosteric regulation is likely to be important. Perhaps consistent with this, Figure 4 suggests that our simulation matches the experimental data less well at the 12 h time point. Thus the differences in both redox and allosteric control of the *O. tauri *enzymes likely contribute to the pre-eminent role of transcript regulation over much of the day. Including post-translational regulation in future models might further improve the model's performance at the light-dark transitions.

In *Ostreococcus *cell division at the end of the light phase seems to dictate the local time of elongation and division of the starch granule and, therefore, the overall timing for the starch synthesis and degradation cycle [23,27,38]. In *C. reinhardtii *the cell cycle is also clearly gated by the circadian clock [43], influencing starch content oscillations rather than the light-dark transition [20]. Regulation of the storage polysaccharide metabolism by the cell cycle has also been reported for *Saccharomyces cerevisiae *[44]. This might be one of the possible explanations for the strong connectivity of genetic regulation and metabolic function observed in our work. The mechanism of starch partitioning and division coinciding with plastid division, as well as the ratio between the chloroplast volume and the volume of the cell, seem to be unique attributes of unicellular green algae.

It has been proposed recently that in addition to the transcriptional regulation of the cell division cycle [38], many other biological processes are globally regulated at the transcriptional level in *Ostreococcus *[27]. Thus, *O. tauri *would be a valuable system for modelling metabolic networks based on transcriptomic data.

The modelling approach presented requires further development before it will be applicable in detail to any plant system, due to the strong differences in the degree of post translational regulation and redox regulation between higher plans and unicellular algae. It is, however, applicable to the systems of similar complexity, such as *Chlamydomonas*, *Micromonas*, or other unicellular algae, which are becoming of great interest as unicellular factories to produce metabolite of interest such as lipids for biofuels. Furthermore, in the absence of detailed kinetic information about the regulation of a metabolic network, such an approximate phenomenological model could be a useful tool for suggesting genetic-metabolic interactions.

## Methods

### Microarray data acquisition and analysis

Microarray design and analysis have been previously described [27,38]. Briefly, genome-wide based Ostreococcus slides (24 K) were manufactured in the Genopole Ouest Transcriptome Platform (Rennes, France) on the basis of 8056 spotted gene-specific 50-mer oligonucleotides (January 2006 annotation, http://bioinformatics.psb.ugent.be/blast/public/?project=ostreococcus). 6369 genes were represented by at least one probe. Cell culture conditions, RNA extraction, labelling, hybridization and raw analysis have been previously described [38]. Normalization was performed using the print-tip loess method and scaled with the Gquantile method. Time courses of gene expression were performed in triplicate, over 27 h, at 3 h intervals (nine time points per time course). Hybridization robustness was checked by performing a hierarchical clustering on the 8041 selected probes using TiGRMeV4.0 suite [45]. Technical triplicates were clustered. Therefore, for further analysis, the median value of each technical triplicate was used. Analysis of Variance (ANOVA) identified 6822 genes differentially expressed with a P value < 10^-3 ^using a 3 factor (genes, biological kinetics and biological replicates) ANOVA. Genes in the final KOG based genome annotation (June 2006) corresponding to *O. tauri *probe sequences were also searched (BLASTX) against the *Arabidopsis *non redundant database to identify the closest orthologue of *Arabidopsis*. Putative genes involved in starch metabolism were identified in the dataset based on the annotations, but also on protein sequence conservation (BLASTP). The complete dataset is available in the Gene Expression Omnibus (GEO) public database at NCBI under the accession number: GSE16422. (Processed data: http://www.ncbi.nlm.nih.gov/geo/query/acc.cgi?token=jbmhpwkyccmuwvu&acc=GSE16422).

### Model building and FBA

The model was developed in COPASI [46], converted to SBML format and analysed with the COBRA 1.3.3 Toolbox for Matlab [47]. This software allows the simulation of maximum starch accumulation using flux-balance analysis (FBA) [48]. Given the CO_2 _uptake rate and the defined starch composition, the maximum possible rate of starch synthesis can be predicted *in silico*. FBA is based on linear optimization of the objective function.

The stoichiometric matrix for the system is of size 38 substrates x 69 reactions. The vector ***v ***has 69 fluxes, including 6 exchange fluxes (b1-b6):

$$v={\left[\text{v1 v2 }...\text{ v56 b1 b2}...\text{ b6}\right]}^{T}$$

Since a starch polysaccharide has an enormous molecular mass, the normal flux value for starch production will be in the range of 10^-5^-10^-6 ^mmol/h, which is difficult to analyse, as it is prone to rounding errors during computations. To avoid this problem, we set all the boundaries for our model reaction rates as a proportion of the maximum starch production rate. This enables us to operate with a solution space in which most of the flux values are greater than one.

#### The objective function

We used linear optimization [47,49] for finding solutions of interest within bounded null space, defined by:

$S_{exch}\left(\begin{array}{c} v\\ b \end{array}\right)=0$, where *v*_*i*,min _ ≤ *v_i _*≤ *v*_*i*,max_, and *b*_*i*,min _ ≤ *b_i _*≤ *b*_*i*,max_

A linear objective function to define has a form:

$$Z=w*\left(\begin{array}{c} v\\ b \end{array}\right)=\sum_{i}w_{i}v_{i}+\sum_{j}w_{j}b_{j},$$

where **w **is a vector of weights (*w_i_*) on the internal flux (*v_i_*) and the external flux (*b_j_*). **Z **then has to be minimized (or maximized as a reciprocal value), depending on the problem posed. Because equations are linear, the *linear programming *(LP) method has been used for this constrained-based optimisation problem.

In the initial setup when the upper bound was defined as a ratio of maximum starch production, the model could either produce starch (if optimized to maximum), or maltose (optimized to minimum). To make the model work in both directions (produce and degrade the starch) simultaneously, with starch production dominating during the day, we introduced the specific cost components for the maltose and glucose transporters and adjusted the upper bounds for the respective reactions appropriately.

Since we aim to model the diurnal cycle in starch production and degradation, and having in mind that starch is generally produced during the day when the carbon source is available, we used the step function (+1 during the day and -1 at night) to fit the starch production rate by simultaneous modification of the maltose transport rate upper bound and the coefficient of that reaction in the cost function. The optimum has been achieved with the values for the export reactions' upper bounds equal to 166.5 and 122.45 for maltose and glucose respectively, which is slightly less than the respective stoichiometric values. Therefore, we assume a kind of competing relationship between starch production and maltose export for carbon fixed by the photosynthetic machinery, with a given privilege for starch synthesis under light exposure.

We introduced light into the system by equating the weight coefficient for the CO_2 _uptake flux in the objective function to one. As a result, nightfall could be easily simulated by switching it to zero, assuming saturating light.

The resulting general linear objective function includes the CO_2 _fixation, starch production, and glucose and maltose export from the chloroplast:

$$Z=1*CO2+10468.135*starch_{ex}+963.325*maltose_{out}+650*glu_{out}$$

To summarize, the stoichiometric analysis presented above gives us the general representation of the capacity of the system and potential variability of its behaviour, depending on applied constraints. In particular, the extreme pathway analysis sheds light on how the system may behave if it is not constrained by the objective function. The sampling of the solution space and, especially, the robustness analysis are extremely useful as they both provide the idea for the each reaction's "importance" in terms of the overall system performance and uncover the existing relationships between the reactions. The latter should be taken into account in experimental design.

Details of analysis are presented in Additional file 4 (Part 2).

### Investigation of timing

All reactions in our model are gene associated. This means that each enzyme responsible for the certain reaction has one or several genes whose function has been annotated correspondently. Each gene has a characteristic pattern of diurnal expression that can be correlated to its specific physiological function.

#### Application of microarray data to flux analysis

We assume that all regulatory feedback influences will be equilibrated rapidly enough to treat the flux system as being in a quasi-steady state on a time scale of three hours. Thus, the behaviour of the system of interest could be approximated as a set of steady states with rapid transitions between them. These steady-states will correspond to the genetic states at different time points available through the microarray data.

Variation of gene expression could lead to a deviation of the reaction flux upper bounds from their value defined by the stoichiometry of the system. To represent such a deviation we scaled RNA abundance from the microarray data and multiplied the flux upper bound by the value obtained. We have chosen the simplest method, which gives us a value in a range between 0 and 1, thus assuming that at the lowest point flux through reaction is blocked, while at the highest level of expression flux is saturated. We understand that aforementioned approximation is quite strong and requires further theoretical investigation, but we have shown in this paper that application of such assumption allowed direct incorporation of gene expression time series into FBA model and, together with analysis of other mechanisms of starch metabolism regulation, described time course of starch content.

Thus, we represent the dynamics of starch content as a set of steady states corresponding to genetic states available through microarray data. Each steady state corresponds to a certain time point of the 12/12 light-dark cycle with an interval of 3 h between the neighbouring points. For those genes from the list with expression profiles that are not available we set the RNA abundance to the minimum level of 0.01 at each time point as there is no reaction controlled by unavailable gene only. For cases where we have more than one gene associated with the same reaction we sum up the gene data for each reaction and multiply the current reaction upper flux bound by the obtained sum.

As mentioned above, expression profiles of some genes involved in starch production and generation are not correlated with a starch dynamics itself. Generally, such discrepancy is explained by post-transcriptional and posttranslational regulation of the protein and metabolic regulation within the pathway. We have shown that in some cases, like O. tauri, when transcriptional regulation has a pronounced effect [27] while the redox regulation of main steps of starch is attenuated, our method suits for illumination of roles of individual enzymes and their timing in resulted orchestration of the systems behaviour

### Ostreococcus starch content analysis

Ostreococcus cultures grown in natural sea water supplemented with Keller enrichment [50] were split-entrained for two weeks under LD: 12/12 cycles (20 μmol quanta.m^-2^.sec^-1^). In these conditions cells are synchronised and achieve one division per day. Cells were inoculated at low density (4 × 10^5 ^cell/ml and 8 × 10^5 ^for cells grown in antiphase with the day/night cycle), 400 ml were dispatched in flasks and further grown for 4 days to reach the approximate density of 3 × 10^6 ^cell/ml and 6 × 10^6 ^cell/ml for antiphase grown cells). Pluronic acid (F-68, sigma, 0.1%) was added to 800 ml of cells that were collected by 8000 g, 8 min, 4°C centrifugation [50] every 3 hours. Cells were completely disrupted by sonication (1 ml, Sodium acetate, 50 mM, pH7.5, 0.5% tritonX100). Cell-extracts were centrifuged (12000 g, 15 min), and white pellets containing insoluble starch were washed twice in water, air dried and kept at 4°C until starch measurement. Starch amounts were measured using the standard amyloglucosidase assay procedure described in [51].

## Authors' contributions

OS designed and performed the modeling, analyzed data and wrote the paper; FC and DD designed and performed experiments, participated in interpretation of results and drafting the manuscript; AS contributed to model design and data analysis; FYB contributed to the design of the study and the experiments, participated in interpretation of results and drafting the manuscript;

SB, IG and AJM contributed to the design of the study and revised the paper. All the Authors read and approved the final manuscript.

## Supplementary Material

Additional file 1 **Figure S2**. The optimal flux distribution for the objective function. Figure S3 and 4 Results for flux variability and robustness analysis. Figure S5 Some results of sampling flux distribution. Figure S6 Transcripts encoding the enzymes of starch metabolism in *O. tauri*. Figure S7 Investigation of effect of single gene rhythmic expression on the overall diurnal starch pattern. Figure S8 Simulation of effect of flux upper bounds constraining on the resulting starch flux pattern. Figure S9 The log flux distribution at ZT0, ZT3, ZT6 and ZT9. Figure S10 The log fluxes distribution at ZT12, ZT15 and ZT18. Figure S11 The weekly pattern of the starch content dynamics. Figure S12 The effect of the *in silico *single gene deletion on the pattern of diurnal maltose production. Figure S13 GWD1 (alpha glucan, water dikinase) from *A. thaliana*, S. *tuberosum*, *C.reinhardtii*, *O.tauri*, *O.lucimarinus *, *M.pusilla *and *M.CCMP490*. Figure S14. Multiple alignment for Beta-amylases from *Arabidopsis, Maize, Brassica*, *C.reinhardtii*, *O.tauri*, *O.lucimarinus* Click here for file

Additional file 2 **Figure S1**. Full map for the starch metabolic pathway considered in the flux balance model. Click here for file

Additional file 3 **Table S1**. The starch pathway reactions described in the model. Table S2. Summary of targets for genetic regulation indentified in different analyses. Click here for file

Additional file 4 **Detailed model assumptions and structure**. Flux balance analysis. Evidence of the moderate redox regulation of starch pathway in *O. tauri*. Click here for file
