# Supplementary material for: Microarray data can predict diurnal changes of starch content in the picoalga Ostreococcus
Source: BMC Syst Biol. 2011 Feb 26;5:36. doi: 10.1186/1752-0509-5-36 (PMC3056741; doi:10.1186/1752-0509-5-36)
Supplement: Additional file 1 — Figure S2. The optimal flux distribution for the objective function. Figure S3 and 4 Results for flux variability and robustness analysis. Figure S5 Some results of sampling flux distribution. Figure S6 Transcripts encoding the enzymes of starch metabolism in O. tauri. Figure S7 Investigation of effect of single gene rhythmic expression on the overall diurnal starch pattern. Figure S8 Simulation of effect of flux upper bounds constraining on the resulting starch flux pattern. Figure S9 The log flux distribution at ZT0, ZT3, ZT6 and ZT9. Figure S10 The log fluxes distribution at ZT12, ZT15 and ZT18. Figure S11 The weekly pattern of the starch content dynamics. Figure S12 The effect of the in silico single gene deletion on the pattern of diurnal maltose production. Figure S13 GWD1 (alpha glucan, water dikinase) from A. thaliana, S. tuberosum, C.reinhardtii, O.tauri, O.lucimarinus , M.pusilla and M.CCMP490. Figure S14. Multiple alignment for Beta-amylases from Arabidopsis, Maize, Brassica, C.reinhardtii, O.tauri, O.lucimarinus [file 1752-0509-5-36-S1.PDF]

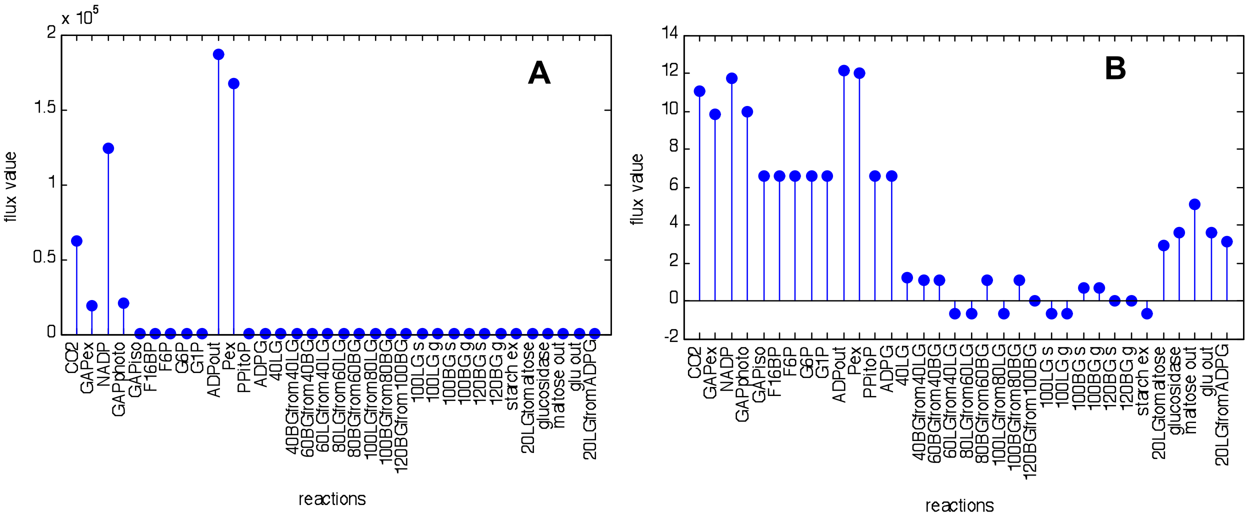

Figure 2 The optimal flux distribution for the objective function (A) The $\log_{10}$ representation is more informative as the distribution of flux values covers several orders of magnitude (B)

Fig.3 Using FVA, minimum and maximum allowable flux values were estimated for the model.

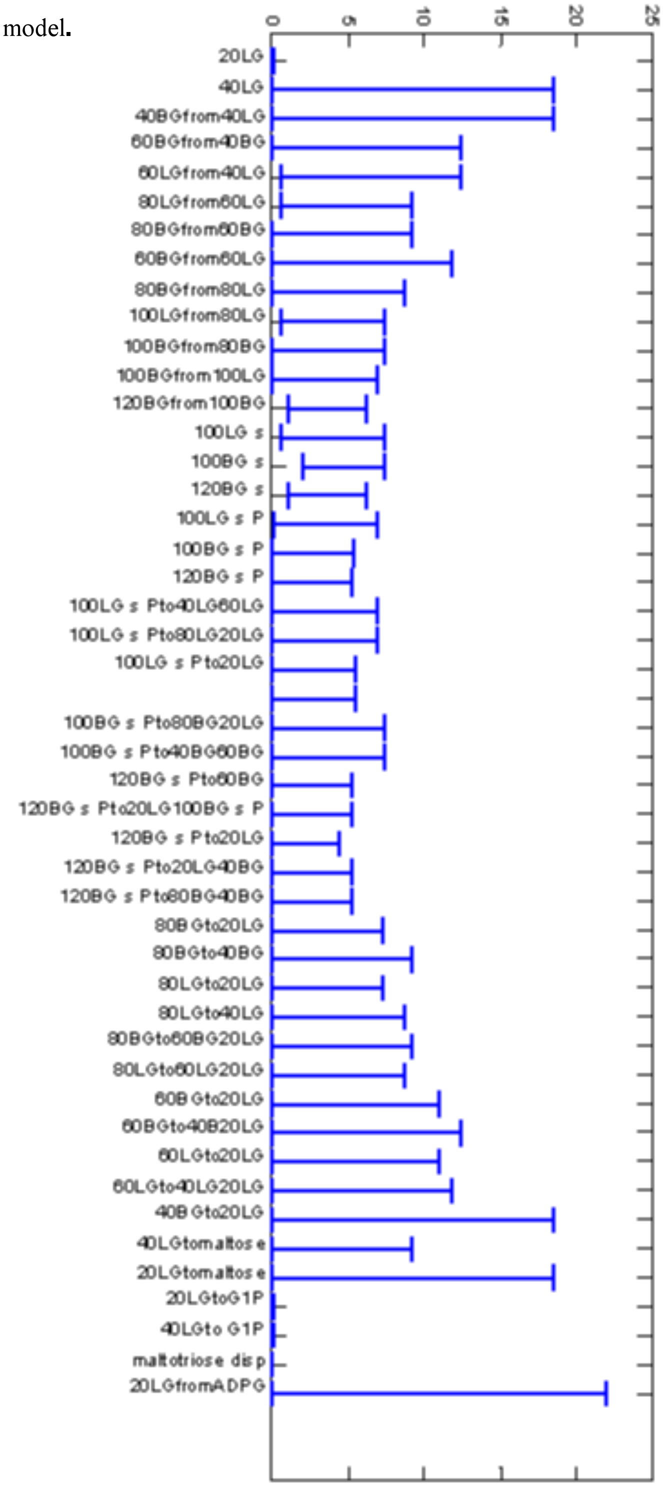

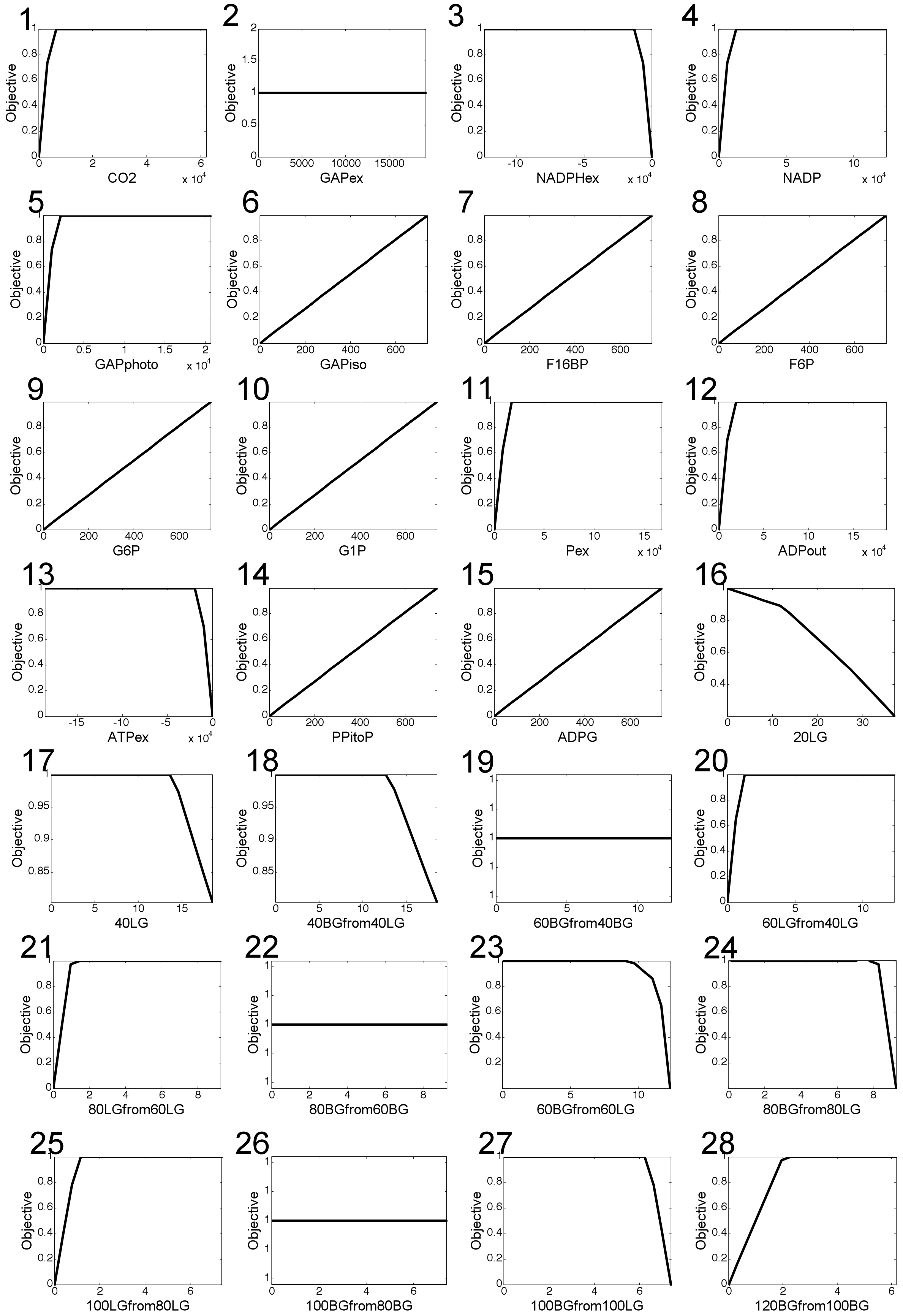

1
Objective
CO2
x 10^4
2
Objective
GAPex
3
Objective
NADPHex
x 10^4
4
Objective
NADP
x 10^4
5
Objective
GAPphoto
x 10^4
6
Objective
GAPiso
7
Objective
F16BP
8
Objective
F6P
9
Objective
G6P
10
Objective
G1P
11
Objective
Pex
x 10^4
12
Objective
ADPout
x 10^4
13
Objective
ATPex
x 10^4
14
Objective
PPitoP
15
Objective
ADPG
16
Objective
20LG
17
Objective
40LG
18
Objective
40BGfrom40LG
19
Objective
60BGfrom40BG
20
Objective
60LGfrom40LG
21
Objective
80LGfrom60LG
22
Objective
80BGfrom60BG
23
Objective
60BGfrom60LG
24
Objective
80BGfrom80LG
25
Objective
100LGfrom80LG
26
Objective
100BGfrom80BG
27
Objective
100BGfrom100LG
28
Objective
120BGfrom100BG

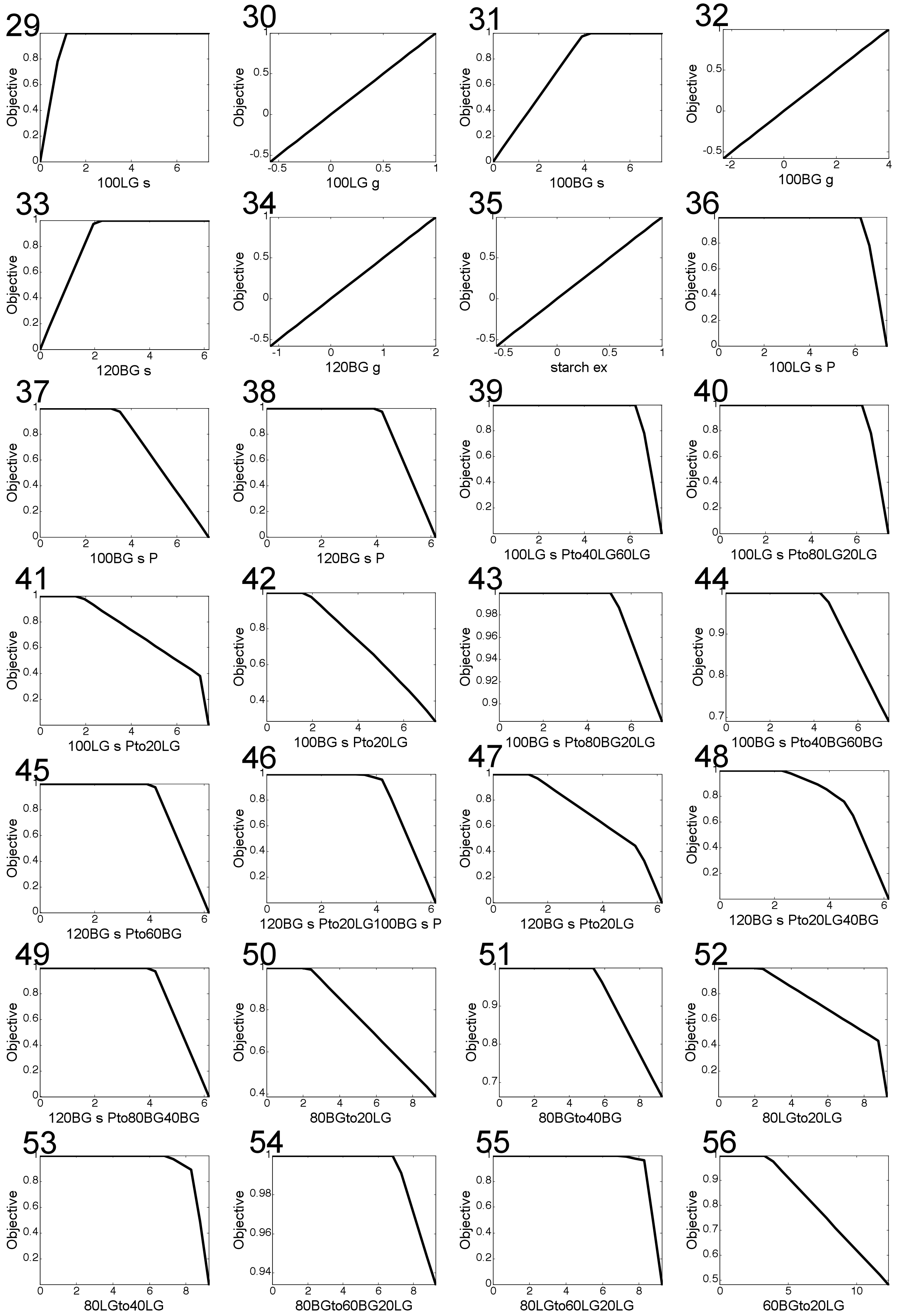
29
Objective
100LG s
30
Objective
100LG g
31
Objective
100BG s
32
Objective
100BG g
33
Objective
120BG s
34
Objective
120BG g
35
Objective
starch ex
36
Objective
100LG s P
37
Objective
100BG s P
38
Objective
120BG s P
39
Objective
100LG s Pto40LG60LG
40
Objective
100LG s Pto80LG20LG
41
Objective
100LG s Pto20LG
42
Objective
100BG s Pto20LG
43
Objective
100BG s Pto80BG20LG
44
Objective
100BG s Pto40BG60BG
45
Objective
120BG s Pto60BG
46
Objective
120BG s Pto20LG100BG s P
47
Objective
120BG s Pto20LG
48
Objective
120BG s Pto20LG40BG
49
Objective
120BG s Pto80BG40BG
50
Objective
80BGto20LG
51
Objective
80BGto40BG
52
Objective
80LGto20LG
53
Objective
80LGto40LG
54
Objective
80BGto60BG20LG
55
Objective
80LGto60LG20LG
56
Objective
60BGto20LG

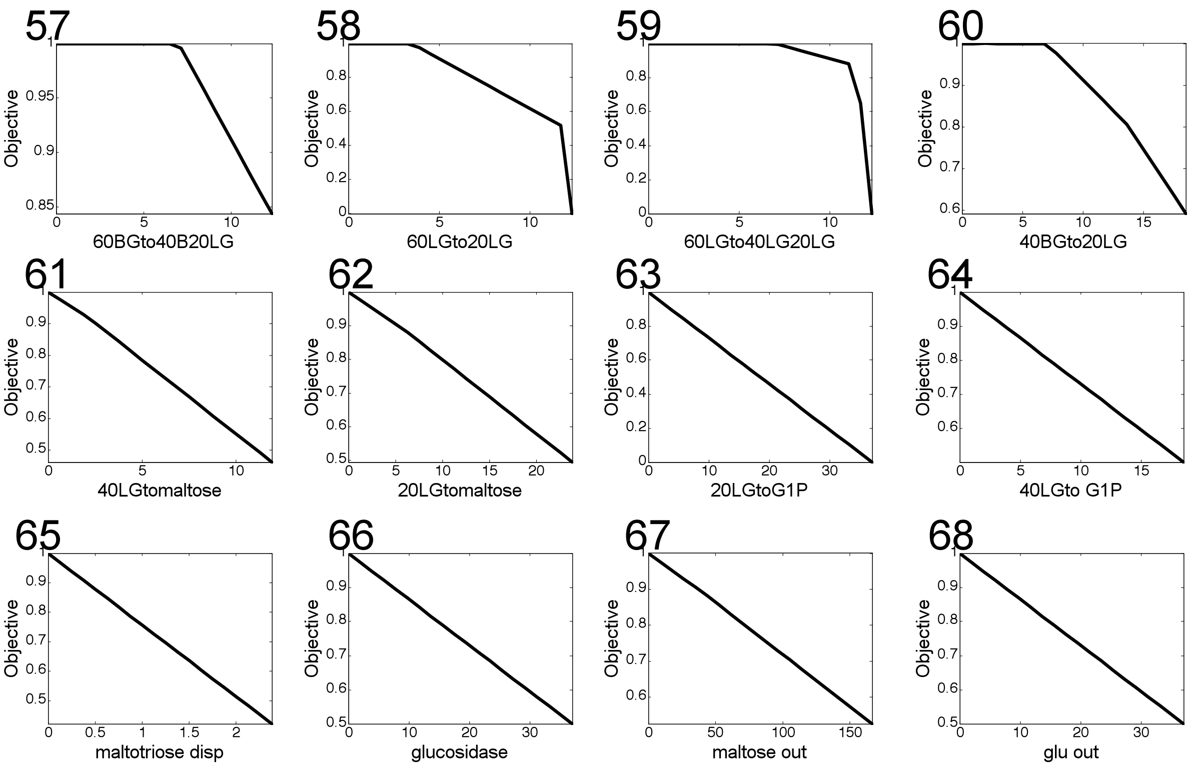

Fig. 4 Robustness analysis. The predicted optimal starch production rate as a function of the flux through each of 69reactions.

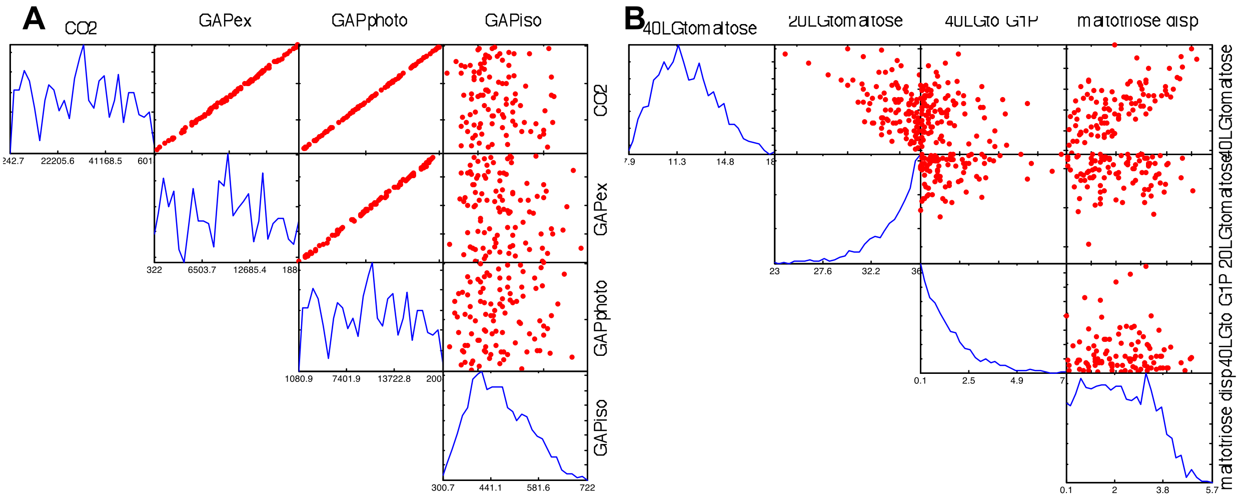

Figure 5 Some results of sampling flux distribution. Flux distribution histograms (diaonal) and pairwise scatterplots (off diagonal) for 4 ininitial and 4 final reactins of the model pathway (reaction names are the same as in Fig G.1) X axis corresponds to the magnitude of the flux through the particular reaction. The off- diagonal scatterplots show the relationships between fluxes through two reactions.

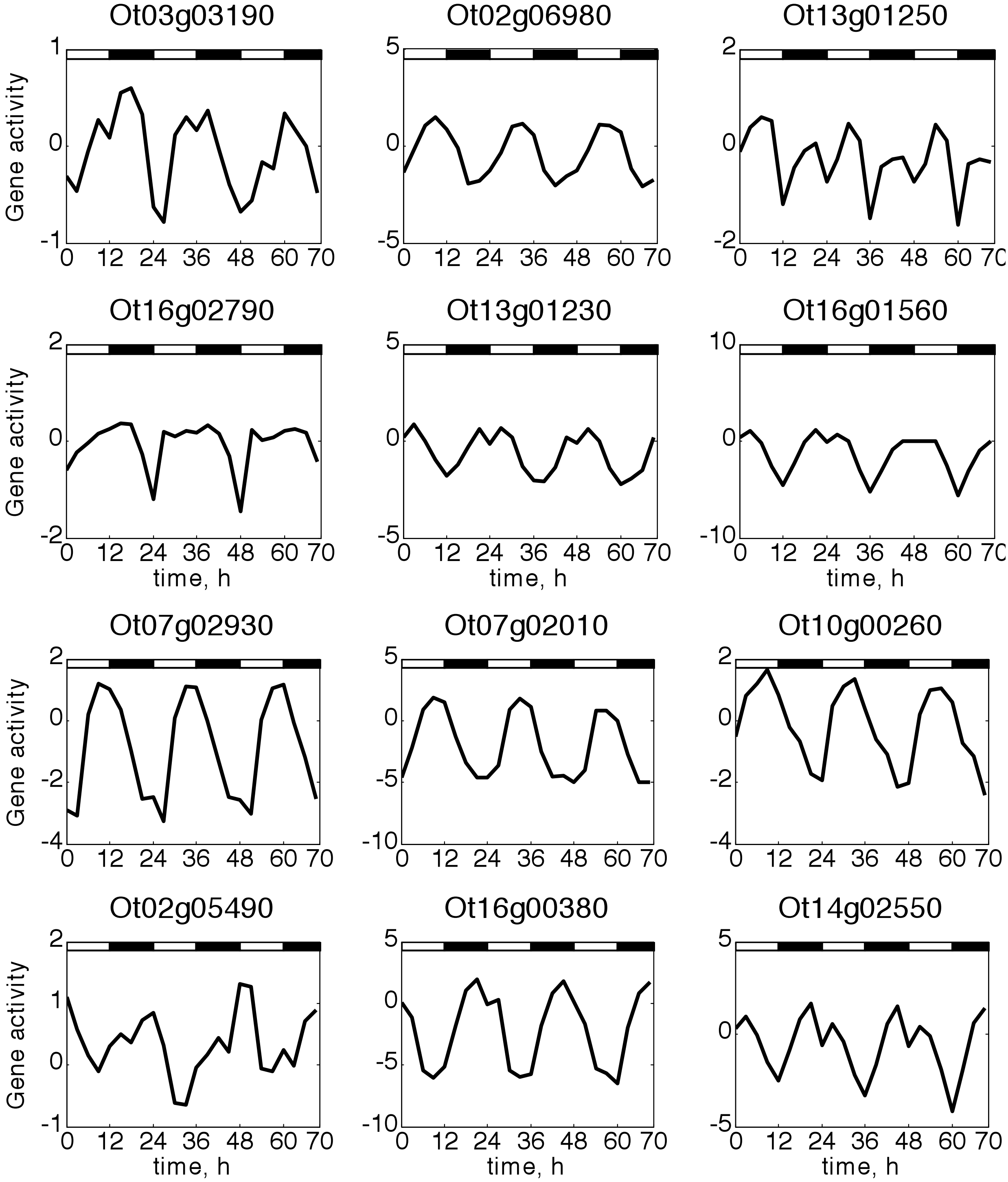
Ot03g03190
Ot02g06980
Ot13g01250
Ot16g02790
Ot13g01230
Ot16g01560
Ot07g02930
Ot07g02010
Ot10g00260
Ot02g05490
Ot16g00380
Ot14g02550
Gene activity
time, h
0
12
24
36
48
60
70

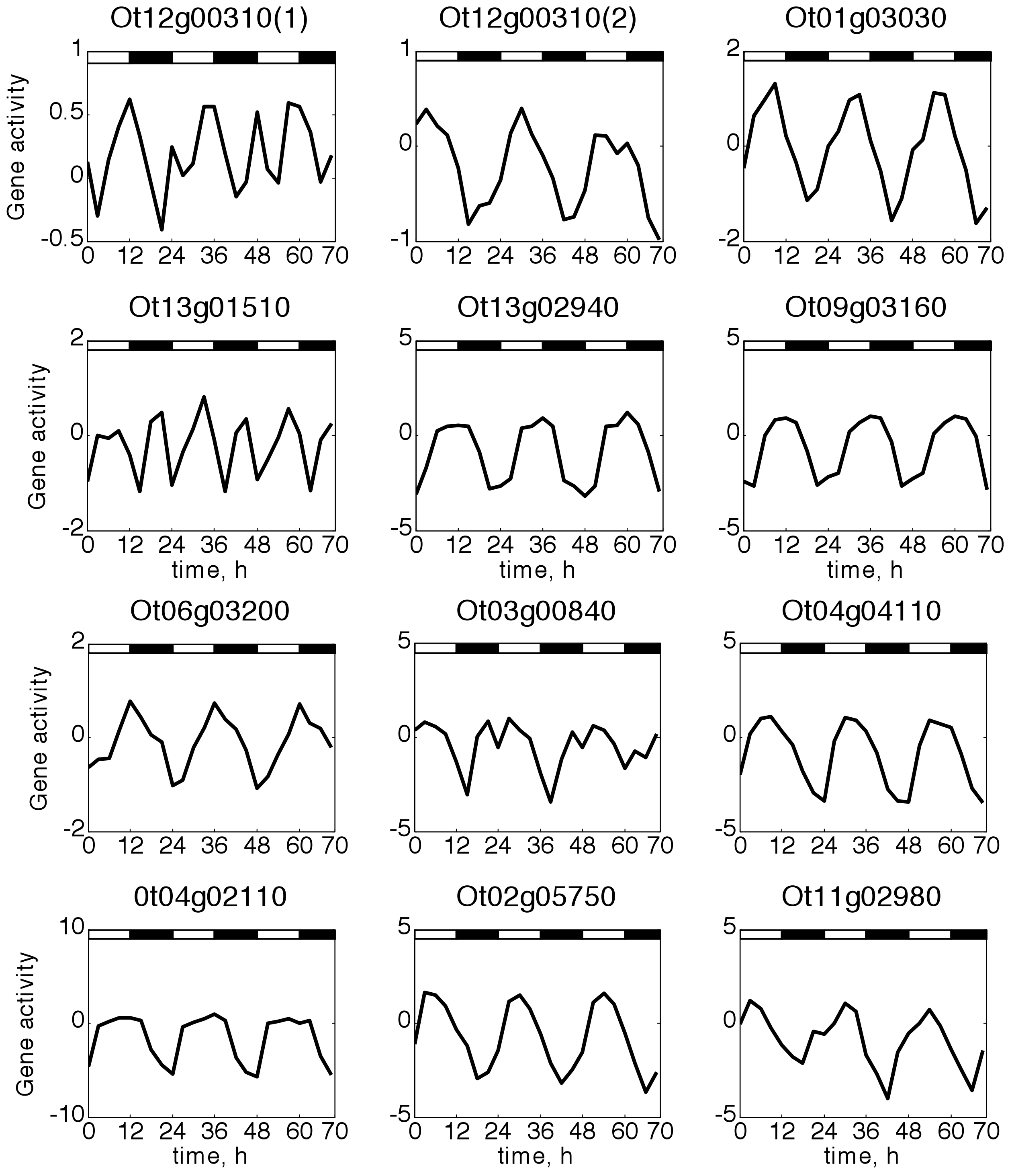

Figure 6 Transcripts encoding the enzymes of starch metabolism in O. tauri.

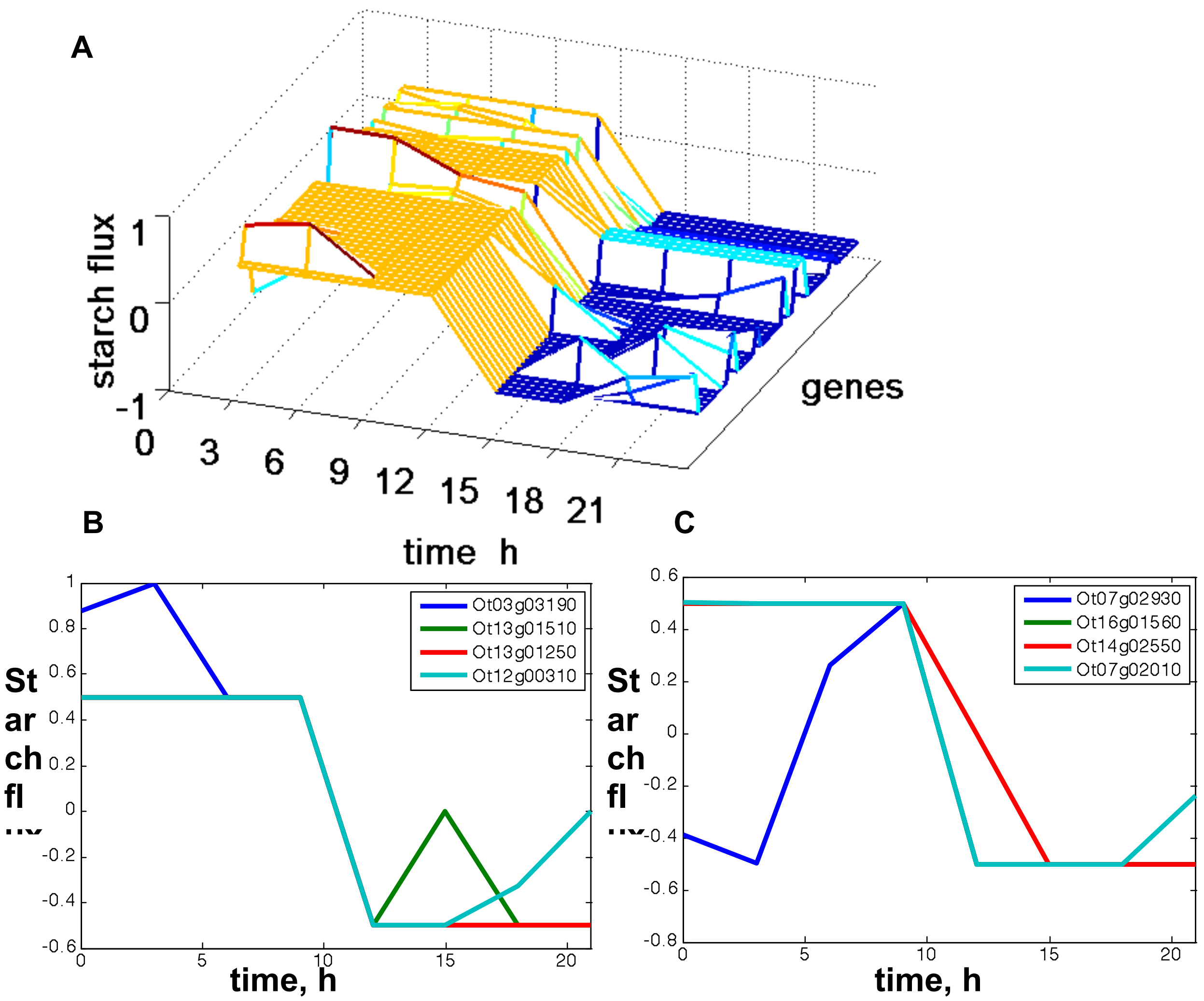

Figure 7. Investigation of effect of single gene rhythmic expression on the overall diurnal starch pattern. Time 0 corresponds to the light onset, time 12 - to the dark onset. A. 3D representation of each gene impact to the shape of the diurnal starch flux. Shown is the null hypothesis: all genes except one are at most expressed. Note time pointes 1 to 8 shown from right to left for the purposes of visualization. B. The effect of expression pattern of Ot03g03190 (beta-amylase), Ot03g01510 (glucan water dikinase) and Ot12g00310 (isoamylase) on the overall starch shape. Ot13g01250 corresponds to the unperturbed shape of the step-function. C. The effect of Ot07g02930 (AGPase), Ot14g02550 (isoamylase). Ot7g02010 and Ot16g01560 correspond to the unperturbed step-function.

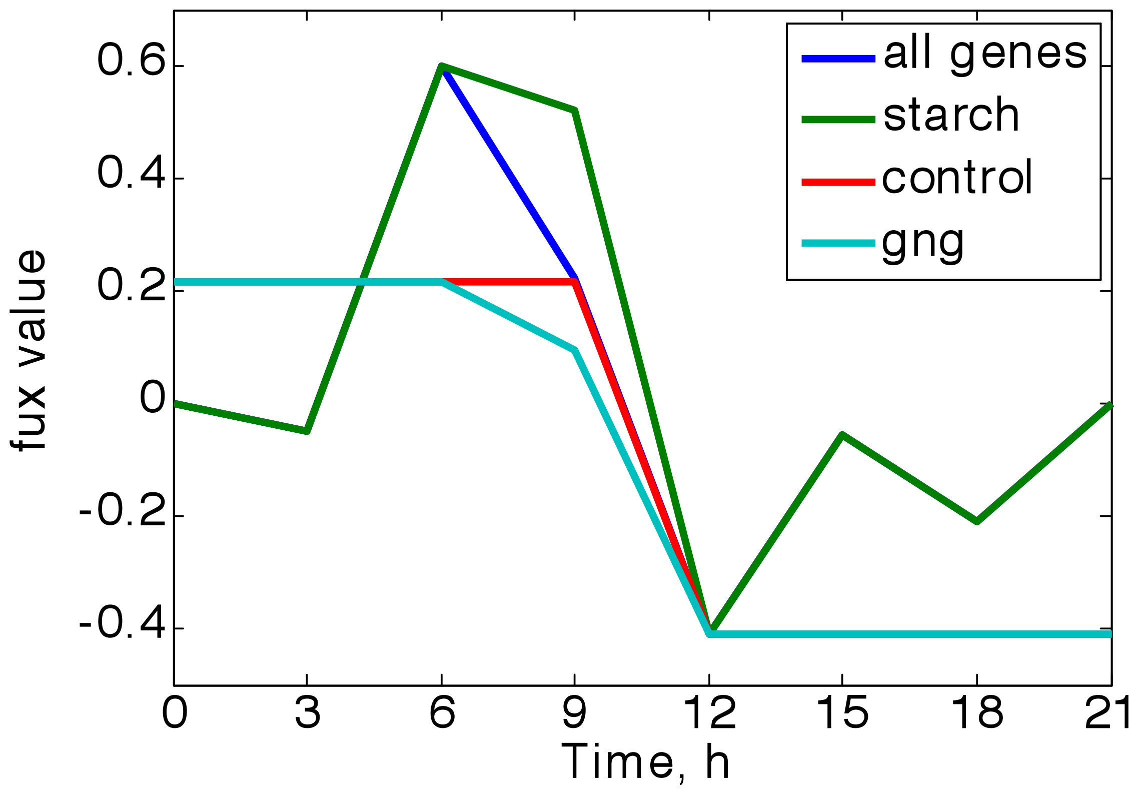

Figure 8 Simulation of effect of flux upper bounds constraining on the resulting starch flux pattern.

Control (red) represents unconstrained case: all the flux upper bound values set to their maximum value (unit); Gng (light blue): the flux upper bounds for reactions for gluconeogenesis are constrained, the rest set to the maximum value.

Starch (green): the flux upper bounds for the starch pathway reactions are constrained, reactions for qluconeogenesis set to the maximum value; all genes (dark blue): all reactions flux upper bounds are constrained.

Constraining of flux upper bounds four reactions for gluconeogenesis influences the starch flux value from 6 to 12h.

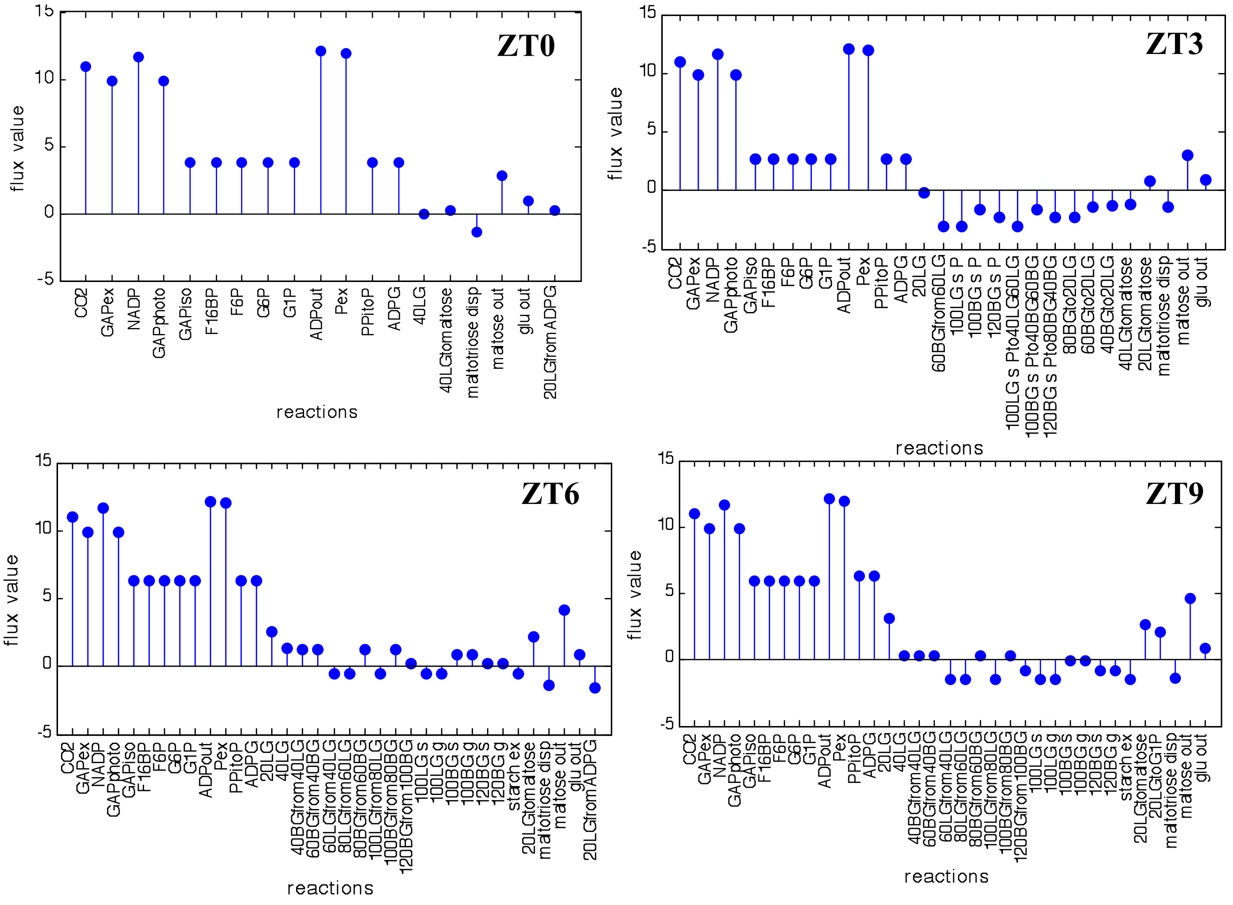

Figure 9 The log flux distribution at ZT0, ZT3, ZT6 and ZT9 (only non-zero fluxes re presented)

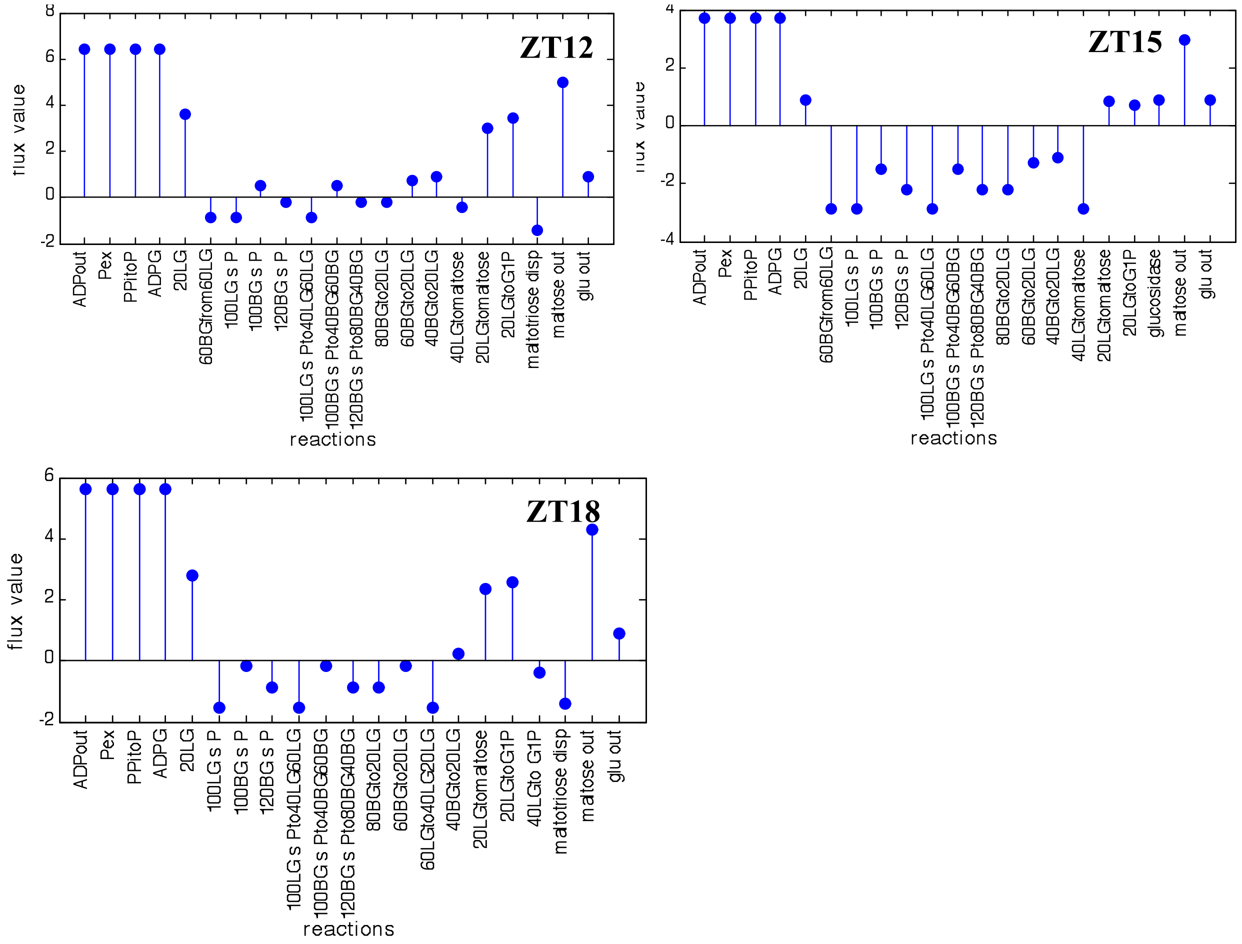

Figure 10 The log fluxes distribution at ZT12, ZT15 and ZT18 (only non-zero fluxes are presented

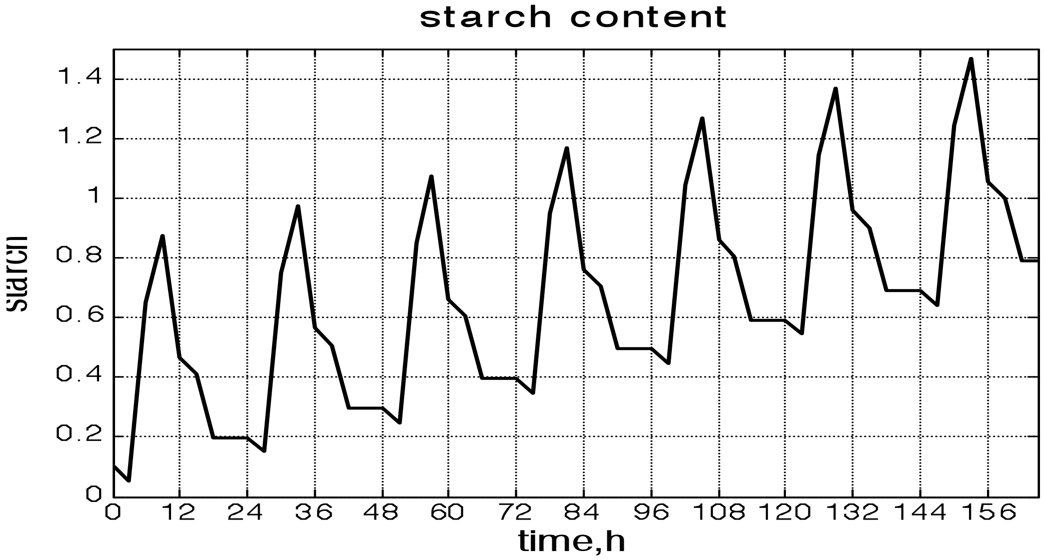

Figure 11. The weekly pattern of the starch content dynamics.

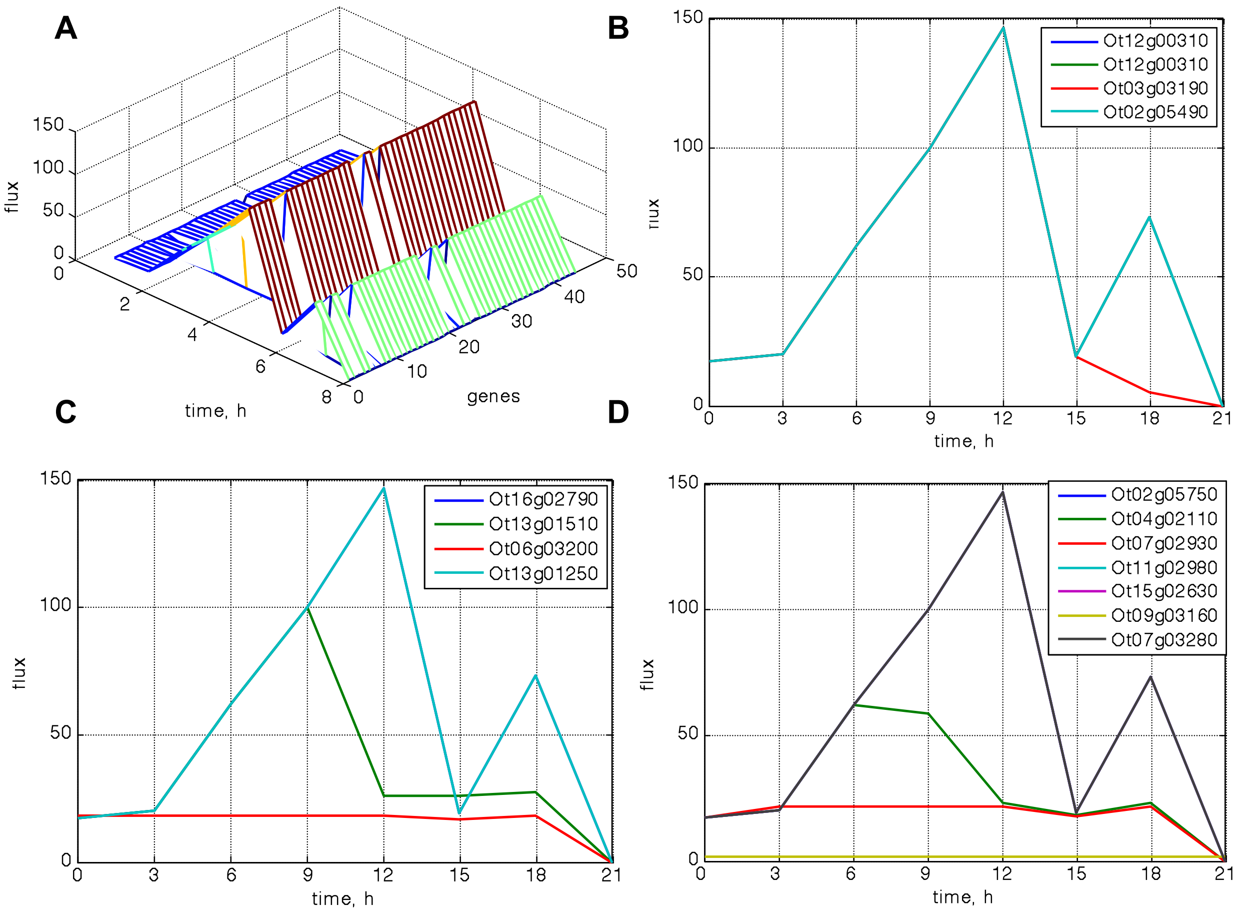

Figure 12 The effect of the *in silico* single gene deletion on the pattern of diurnal mltose production. A. The 3D representation of the effect of single gene deletion on the shape of the maltose diurnal pattern. B. The effect of the deletion of the Ot03g03190 (beta-amylse) on the maltose diurnal pattern. Ot12g00310 corresponds to the unperturbed shape. C. The effect of the deletion of the Ot06g03200 (GBSSI) and Ot13g01510 (glucan, water dikinase) on the shape of the maltose diurnal pattern. Ot13g01250 correspond to the unperturbed shape. D. The effect of the deletion of the Ot07g02930 (AGPase), Ot0402110 (starch phosphorylase), Ot09g03160 (MEX1) on the shape of the maltose diurnal pattern. Ot07g03280 correspond to the unperturbed shape.

Arabidopsis_thaliana_(GWD1)/1-1399	992	AVLTPDMPDVLSHVSVRARNGKICFATCFDSGILS	1026
Citrus_reticulata_(R1)/1-1475	1065	AVLTADMPDVLSHVSVRARNCKVCFATCFDPNILA	1099
Solanum_tuberosum_(R1)/1-1464	1057	ALITPDMPDVLSHVSVRARNGKVCFATCFDPNILA	1091
Solanum_lycopersicum_(GWD)/1-1465	1058	ALITPDMPDVLSHVSVRARNGKVCFATCFDPNILA	1092
Arabidopsis_thaliana_(GWD2)/1-1278	874	AVLTPSMIDVLSHVSIRARNSKACFATCFDQNVLS	908
Ostreococcus_tauri_(R1-C)/1-1079	669	AVVTRAPVDLLSHIAIRARNTNVLLASVSDDALWG	703
Ostreococcus_tauri_(spr1b)/1-1612	1191	AIITPDMPDILSHCAVRARNEKVLFATAFDVSMFE	1225
Ostreococus_lucimarinus/1-1411	989	AIVTPDMPDVLSHCAVRARNEKVLFATLFDVNVLE	1023
Micromonas_sp._RCC299/1-1419	990	GLITPDMPDVLAHTSVRARNERVLFATVFDAGRMS	1024
Micromonas_pusilla_CCMP1545/1-1562	1122	GVITPDMPDILSHVSVRARNEGCLFATVFDAGKLQ	1156

Figure 13. GWD1 (alpha glucan , water dikinase) from *A. thaliana*, S. *tuberosum*, *C.reinhardtii*, *O.tauri*, *O.lucimarinus* , *M.pusilla* and *M.CCMP490*. The sequence CFATC responsible for disulfide bond formation could not be found in unicellular green algae.

Aravidopsis_thaliana_(TR-BAMY)/1-575	53	DPSPPMSPILGATRADLSVACKAFAVENG-------I	82
Brassica_napus/1-569	53	NVSPPMSPVLGSRRADLSVACKAFAVE----------	79
Chlamydomonas_reinhardtii/1-594	75	EPIVTTPGASAFSEVEDDLQIHRLLRETGQHIKIPMI	111
Ostreococcus_lucimarinus/1-480		-------------------------------------	
Micromonas_pusilla_CCMP1545/1-546		------------------------------------M	1
Micromonas_sp._RCC299_/1-465		-------------------------------------	
Ostreococcus_tauri_(Bamy2)/1-365		------------------------------------M	1
Ostreococcus_tauri_(Ot02g06980)/1-458		-------------------------------------	
Aravidopsis_thaliana_(TR-BAMY)/1-575	475	AGENALPRYDDYAHEQILKASALNLDQNNEGEPRE--	509
Brassica_napus/1-569	469	AGENALPRYEDYAHEQILKASALSFDQNSEGENRE--	503
Chlamydomonas_reinhardtii/1-594	534	SGENALQRYDDYAFERIAESAFGRNARAGR-------	563
Ostreococcus_lucimarinus/1-480	376	AGENALCRFDQAAYDKIIKNCAGEGNDEEMWREGTML	412
Micromonas_pusilla_CCMP1545/1-546	420	AGENALCRFDQDAYDKIITNCRGEGNESARWESGALL	456
Micromonas_sp._RCC299_/1-465	398	SAENALYRCDSGAYKQMVRNSMGLSGDG---GGG---	428
Ostreococcus_tauri_(Bamy2)/1-365	264	AGENALCRFDQVAFDKIIKNCAGEGNDEEMWREGTIL	300
Ostreococcus_tauri_(Ot02g06980)/1-458	354	TENALARFDADAYAQILRAYERHGAATMAATTASEDA	390
Aravidopsis_thaliana_(TR-BAMY)/1-575	510	--MCAFTYLRMNPELFQADNWGKFVAFVKKMGEGRDS	544
Brassica_napus/1-569	504	--MCAFTYLRMNPELFKADNWGKFVGFVKKMGEGRDS	538
Chlamydomonas_reinhardtii/1-594	564	--LTQVTFLRMGDLMFD--NWDAFSRFLNRMRNKA--	594
Ostreococcus_lucimarinus/1-480	413	PPMACFTFLRFNAELFSPFAFESFRIFVQRMRDETGL	449
Micromonas_pusilla_CCMP1545/1-546	457	PPMASFTFLRMTRELFEDDNFNSFVHFVTRMANETGV	493
Micromonas_sp._RCC299_/1-465	429	--MHSFTFLRLCDSLMEPDNFAQFETFVRDMSGDS--	461
Ostreococcus_tauri_(Bamy2)/1-365	301	PPMACFTFLRFNSELFSPGAFESFRIFVQRMRDETGL	337
Ostreococcus_tauri_(Ot02g06980)/1-458	391	TANEENGSLRSASSDETTAPGSRASFESNRGVRERVL	427

Figure 14. Multiple alignment for A. Beta-amylases from *Arabidopsis, Maize,* Brassica, *C.reinhardtii*, *O.tauri*, *O.lucimarinus* , *M.pusilla* and *M.CCMP490*. Cysteins Sequences for the unicellular green algae do not contain the two cysteins in marked positions. B. GWD1 (alpha glucan, water dikinase) from *A. taliana*, S. *tuberosum*, *C.reinhardtii*, *O.tauri*, *O.lucimarinus*, *M.pusilla* and *M.CCMP490*. The sequence CFATC responsible for disulfide bond formation is absent in unicellular green algae.
