# Supplementary material for: Microarray data can predict diurnal changes of starch content in the picoalga Ostreococcus
Source: BMC Syst Biol. 2011 Feb 26;5:36. doi: 10.1186/1752-0509-5-36 (PMC3056741; doi:10.1186/1752-0509-5-36)
Supplement: Additional file 2 — Figure S1. Full map for the starch metabolic pathway considered in the flux balance model. [file 1752-0509-5-36-S2.PDF]

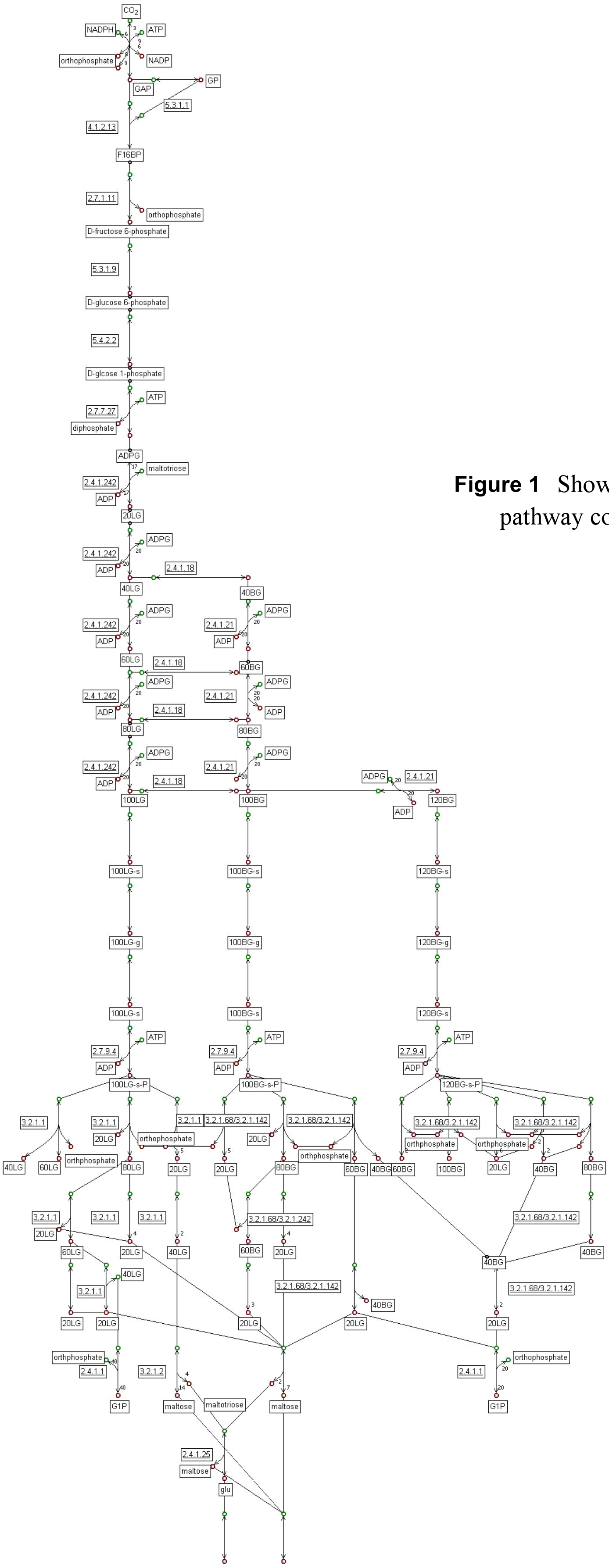

Figure 1 Shown is a full map for the starch metabolic pathway considered In the flux balance model.
