# Supplementary material for: Microarray data can predict diurnal changes of starch content in the picoalga Ostreococcus
Source: BMC Syst Biol. 2011 Feb 26;5:36. doi: 10.1186/1752-0509-5-36 (PMC3056741; doi:10.1186/1752-0509-5-36)
Supplement: Additional file 3 — Table S1. The starch pathway reactions described in the model. Table S2. Summary of targets for genetic regulation indentified in different analyses. [file 1752-0509-5-36-S3.PDF]

Supporting Table 1 The starch pathway reactions described in the model.

reaction name	reaction	ub e6	lb	cost e4	EC
'CO2'	-> CO2	0.08	**0**	0.0001	
'GAPex'	GAP <=>	0.0192	0	0	
'NADPHex'	NADPH <=>	1	-1000000	0	
'NADP'	NADP <=>	1	-1000000	0	
'GAPphoto'	3 CO2 + 6 NADPH + 9 ATP -> GAP + 6 NADP + 9 ADP + 8 P	1	0	0	
'GAPiso'	GAP <=> GP	1	-100000	0	5.3.1.1
'F16BP'	GAP + GP -> F16BP	0.00074	0	0	4.1.2.13
'F6P'	F16BP -> P + F6P	0.00074	0	0	3.1.3.11
'G6P'	F6P -> G6P	0.00074	0	0	5.3.1.9
'G1P'	G6P -> G1P	0.00074	0	0	5.4.2.2
'ATPex'	ATP <=>	1	-1000000	0	
'ADPout'	ADP ->	1	-1000000	0	
'Pex'	P <=>	1	-1000000	0	
'PPitoP'	PPi -> 2 P	1	-1000000	0	
'ADPG'	ATP + G1P -> PPi + ADPG	0.00074	0	0	2.7.7.27
'20LG'	17 ADPG + maltotriose -> 17 ADP + 20LG	0.000037	0	0	2.4.1.242
'40LG'	20 ADPG + 20LG -> 20 ADP + 40LG	1.85E-05	0	0	2.4.1.21
'40BGfrom40LG'	40LG -> 40BG	1.85E-05	0	0	2.4.1.18
'60BGfrom40BG'	20 ADPG + 40BG -> 20 ADP + 60BG	1.23E-05	0	0	2.4.1.21
'60LGfrom40LG'	20 ADPG + 40LG -> 20 ADP + 60LG	1.23E-05	0	0	2.4.1.242
'80LGfrom60LG'	20 ADPG + 60LG -> 20 ADP + 80LG	9.25E-06	0	0	2.4.1.242
'80BGfrom60BG'	20 ADPG + 60BG -> 20 ADP + 80BG	9.25E-06	0	0	2.4.1.21

'60BGfrom60LG'	60LG -> 60BG	1.23E-05	0	0	2.4.1.18
'80BGfrom80LG'	80LG -> 80BG	9.25E-06	0	0	2.4.1.18
'100LGfrom80LG'	20 ADPG + 80LG -> 20 ADP + 100LG	7.40E-06	0	0	2.4.1.242
'100BGfrom80BG'	20 ADPG + 80BG -> 20 ADP + 100BG	7.40E-06	0	0	2.4.1.21
'100BGfrom100LG'	100LG -> 100BG	7.40E-06	0	0	2.4.1.18
'120BGfrom100BG'	20 ADPG + 100BG -> 20 ADP + 120BG	6.17E-06	0	0	2.4.1.21
'100LG s'	100LG -> 100LG-s	7.40E-06	0	0	
'100LG g'	100LG-s <=> 100LG-g	7.40E-06	-1000000	0	
'100BG s'	100BG -> 100BG-s	7.40E-06	0	0	
'100BG g'	100BG-s <=> 100BG-g	7.40E-06	-1000000	0	
'120BG s'	120BG -> 120BG-s	6.17E-06	0	0	
'120BG g'	120BG-s <=> 120BG-g	6.17E-06	-1000000	0	
'starch ex'	100LG-g + 4 100BG-g + 2 120BG-g <=>	1.12E-06	-1000000	1.04681	
'100LG s P'	P + 100LG-s -> 100LG-s-P	7.40E-06	0	0	2.7.9.4
'100BG s P'	P + 100BG-s -> 100BG-s-P	7.40E-06	0	0	2.7.9.4
'120BG s P'	P + 120BG-s -> 120BG-s-P	6.17E-06	0	0	2.7.9.4
'100LG s Pto40LG60LG'	100LG-s-P -> P + 40LG + 60LG	7.40E-06	0	0	3.2.1.1
'100LG s Pto80LG20LG'	100LG-s-P -> P + 20LG + 80LG	7.40E-06	0	0	3.2.1.1
'100LG s Pto20LG'	100LG-s-P -> P + 5 20LG	7.40E-06	0	0	3.2.1.1
'100BG s Pto20LG'	100BG-s-P -> P + 5 20LG	7.40E-06	0	0	3.2.1.68
'100BG s Pto80BG20LG'	100BG-s-P -> P + 20LG + 80BG	7.40E-06	0	0	3.2.1.142
'100BG s Pto40BG60BG'	100BG-s-P -> P + 40BG + 60BG	7.40E-06	0	0	3.2.1.68
'120BG s Pto60BG'	120BG-s-P -> P + 2 60BG	6.17E-06	0	0	3.2.1.68
'120BG s Pto20LG100BG s P'	120BG-s-P -> 20LG + 100BG-s-P	6.17E-06	0	0	3.2.1.68
'120BG s Pto20LG'	120BG-s-P -> P + 6 20LG	6.17E-06	0	0	3.2.1.68

'120BG s Pto20LG40BG'	120BG-s-P -> P + 2 20LG + 2 40BG	6.17E-06	0	0	3.2.1.142
'120BG s Pto80BG40BG'	120BG-s-P -> P + 40BG + 80BG	6.17E-06	0	0	3.2.1.142
'80BGto20LG'	80BG -> 4 20LG	9.25E-06	0	0	3.2.1.68
'80BGto40BG'	80BG -> 2 40BG	9.25E-06	0	0	3.2.1.142
'80LGto20LG'	80LG -> 4 20LG	9.25E-06	0	0	3.2.1.1
'80LGto40LG'	80LG -> 2 40LG	9.25E-06	0	0	3.2.1.1
'80BGto60BG20LG'	80BG -> 20LG + 60BG	9.25E-06	0	0	3.2.1.68
'80LGto60LG20LG'	80LG -> 20LG + 60LG	9.25E-06	0	0	3.2.1.1
'60BGto20LG'	60BG -> 3 20LG	1.23E-05	0	0	3.2.1.68
'60BGto40B20LG'	60BG -> 20LG + 40BG	1.23E-05	0	0	3.2.1.68
'60LGto20LG'	60LG -> 3 20LG	1.23E-05	0	0	3.2.1.1
'60LGto40LG20LG'	60LG -> 20LG + 40LG	1.23E-05	0	0	3.2.1.1
'40BGto20LG'	40BG -> 2 20LG	1.85E-05	0	0	3.2.1.68
'40LGtomaltose'	40LG -> 4 maltotriose + 14 maltose	1.85E-05	0	0	3.2.1.2
'20LGtomaltose'	20LG -> 2 maltotriose + 7 maltose	0.000037	0	0	3.2.1.2
'20LGtoG1P'	20 P + 20LG -> 20 G1P	0.000037	0	0	2.4.1.1
'40LGto G1P'	20 P + 40LG -> 20 G1P + 20LG	1.85E-05	0	0	2.4.1.1
'maltotriose disp'	20LG + 20 maltotriose -> 40LG + 10 glu	0.000247	0	0	2.4.1.25
'glucosidase'	maltotriose -> maltose + glu	0.000247	0	0	3.2.1.20
'maltose out'	maltose ->	0.000167	0	0.096333	MEX1
'glu out'	glu ->	0.000122	0	0.065003	
'20LGfromADPG'	20ADPG -> 20 ADP + 20LG	0.00037	0	0	2.4.1.21

Supporting Table 2 Summery for targets for genetic regulation identified from different analysis

Regulation targets obtained in the robustness analysis (reactions)	Regulation targets obtained in single gene deletion analysis (genes)	Regulation targets obtained in gene overexpression analysis	One array substitution
GAPiso			
F16BP	Ot10g01490 (fructose-bisphosphate aldolase)		
F6P	Ot03g00330 (fructose 1,6 bisphosphotase)		
G6P			
G1P	Ot15g02630 (phosphoglucomutase)		
ADPG	Ot7g02930 (AGPase)		Ot07g02930 (AGPase)
20LG	Ot06g03200 (GBSSI)	Ot06g03200 (GBSSI)	
100LGs_Pto20LG			Ot07g02010 (alpha-amylase)
100BGs_Pto 20LG			Ot12g00310 and Ot14g02550 (isoamylases)
120BGs_Pto20LG			Ot12g00310 and Ot14g02550 (isoamylases)
80BGto20LG			Ot12g00310 and Ot14g02550 (isoamylases)
80LGto20LG			Ot07g02010 (alpha-amylase)
60BGto20LG			Ot12g00310 and Ot14g02550 (isoamylases)
20LGto maltose	Ot03g03190 (beta –amylase)		Ot03g03190 (beta-amylase)
40LFto maltose	Ot03g03190 (beta –amylase)		Ot03g03190 (beta –amylase)
20LGtoG1P	Ot04g02110 (starch phosphorylase)	Ot11g00280 and Ot11g01020 (phophorylase)	
40LGtoG1P	Ot04g02110 (starch phosphorylase)	Ot11g00280 and Ot11g01020 (phosphrylase)	

maltotriose_disp			
glucosidase			
maltose out	Ot09g03160 (MEX1)		
glu out		Ot14g01870 and Ot03g05590 (glucose transprtes)	
	Ot13g01510 (glucan, waterdikinase)	Ot08g01260 (glucan, water dikinase)	Ot03g01510 (glucan water dikinase)
