# Supplementary material for: Microarray data can predict diurnal changes of starch content in the picoalga Ostreococcus
Source: BMC Syst Biol. 2011 Feb 26;5:36. doi: 10.1186/1752-0509-5-36 (PMC3056741; doi:10.1186/1752-0509-5-36)
Supplement: Additional file 4 — Detailed model assumptions and structure. Flux balance analysis. Evidence of the moderate redox regulation of starch pathway in O. tauri. [file 1752-0509-5-36-S4.PDF]

Supporting materials

Part1

Model assumptions and structure

- We consider all the processes described in the model to take place in one compartment (chloroplast) with a limited flux exchange with the cytosol (GAP, ATP, ADP, P, NADP, and NADPH).
- We consider starch to be produced under light conditions from the D-glyceraldehyde 3-phosphate (GAP) obtained by CO_2 fixation during photosynthesis.
- Not all the GAP produced during the photosynthesis is utilized by starch biosynthesis, but about the half of the overall flow. The rest is released to the cytosol, so that for every 100 molecules of CO_2, giving about 33 molecules of GAP, 16 of them go to the cytosol and 17 to starch.
- Starch consists of amylose and amylopectin in the approximate ratio 2:8 [1]. Our model starch consists of long linear glucans (100 glucose residues) and large branched glucans (100 and 120 glucose residues), thus we consider starch as a composite of three main components: 100LG (long linear glucan of 100 glucose residues), 100BG (branched glucan of 100 glucose residues) and 120 BG (branched glucan of 120 glucose residues) in the ratio 1:2:4.

  The exchange reaction for starch formation in our model, thus, has the form of:

100LG-g + 4 100BG-g + 2 120BG-g <=>, that fixes the ratio

- We also take into consideration short linear glucans (20 glucoses), middle linear (40, 60 and 80 glucoses) and middle branched (60 and 80 glucoses). All these species work as intermediate steps during the branching and debranching processes.
- The branching happens without elongation of the chain, just by the rearrangement of the existing linear chain by SBE (2.4.1.18) [2].
- Elongation happens by means of the SS (2.4.1.21) and GBSSI (2.4.1.242) enzymes with ADP-glucose (ADPG) as a building block [3]. We consider the granule-bound starch synthase (GBSSI) working mainly for elongation of linear glucans, as it is known to produce the amylose fraction within the granule (Denyer, 2001). We also allow GBSSI to use the malto-oligosaccharides, produced during the starch degradation, for amylose priming (Denyer, 2001). For simplicity, we do not distinguish between different types of

starch synthase (SSI-SSIV) (Roldan, 2007), specifying their function as elongation of branched glucans comprising the amylopectin fraction.

- Both small glucans (20LG) and glucose-1-phosphate (G1P) are allowed reutilization during the starch synthesis as building components; thus, we get a number of internal cycles within the whole pathway. This assumption is based upon the so called 'glucan-trimming'model of starch synthesis (Ball, 1996, Takaha, 1998, Tetlow, 2004), and enables isoamylases (3.2.1.68/3.2.1.142) and starch phosphorylases (2.4.1.1) to participate in starch granule formation (Watterbled, 2008).
- The disproportionating enzyme (2.4.1.25, D-enzyme) in the model produces the middle linear glucans (40LG) from malto-oligosaccharides (maltotrioses). These middle glucans are also allowed for further branching during the amylopectin synthesis (Colleoni, 1999).
- We assume that the components of the starch granule can be accessed by degradation enzymes only after phosphorylation by alpha-glucan, water dikinase enzyme (GWD) (2.7.9.4) [4].
- Only the components of the granule surface are accessible for phosphorylation followed by subsequent degradation (100LG_s, 100BG_s, and 120BG_s). We consider the starch granule as a separate compartment, and design an exchange of surface granule components with the granule body. Once starch components come to the granule body and are covered by a new layer of surface components, they are no longer available to degradation enzymes (100LG_g, 10BG_g, 120BG_g) (Fig S1, Table S1).

Part 2

Flux balance analysis

In the initial setup when the upper bound was defined as a ratio of maximum starch production, the model could either produce starch (if optimized to maximum), or maltose (optimized to minimum). To make the model work in both directions (produce and degrade the starch) simultaneously, with starch production dominating during the day, we introduced the specific cost components for maltose and glucose transporters and adjusted the upper bounds for the respective reactions appropriately.

Since we aim to model the diurnal cycle in starch production and degradation, and having in mind that starch is generally produced during the day when the carbon source is available, we used the step function (+1 during the day and -1 at night) to fit the starch production rate by simultaneous modification of the maltose transport rate upper bound and the coefficient of that reaction in the cost function. The optimum has been achieved with the values for the export

reactions' upper bounds equal to 166.5 and 122.45 for maltose and glucose respectively, which is slightly less than the respective stoichiometric values. Therefore, we assume a kind of competing relationship between starch production and maltose export for carbon fixed by the photosynthetic machinery, with a given privilege for starch synthesis under light exposure.

We introduced light into the system by equating the weight coefficient for the CO_2 uptake flux in the objective function to one. As a result, nightfall could be easily simulated by switching it to zero, assuming saturating light.

The resulting general linear objective function includes the CO_2 fixation, starch production, and glucose and maltose export from the chloroplast:

$$Z = 1 * CO2 + 10468.135 * starch_{ex} + 963.325 * maltose_{out} + 650 * glu_{out}$$

The optimal flux distribution for the objective is presented below. Only reactions with flux values different from zero are depicted in Supplementary Figure 2.

Flux variability analysis

We determined the range of fluxes that correspond to the optimum solution by means of Flux Variability Analysis (FVA), provided by the COBRA Toolbox [Becker]. The range has been computed for each flux by solving the following LP problems [5],[6]:

maximize or minimize v_i

subject to: $Z = Z_{opt}$

$\mathbf{S} * \mathbf{v} = \mathbf{0}$

$v_{i,\min} \le v_i \le v_{i,\max}$ for i=1,…, n

The results of FVA are presented in Supplementary Figure 3. It is clear from the figure that due to the given objective function those fluxes that contributed to the objective ('CO_2', 'starch_ex', 'maltose_out', 'glucose out') have a fixed value and, accordingly, zero range. The same is true for the linear gluconeogenetic and ADP-glucose producing branch of the pathway ('F16BP', 'F6P', 'G6P', 'G1P', 'ADPG'), as it is strictly constrained by the objective function for a starch synthesis rate set to maximum. Flux values for other components vary depending on how compensated this particular reaction is relative to the whole pathway. For instance, for reactions from the debranching part of the model the range could be noticeably larger than for reactions from the branching part because the combinatorics of debranching options allows substitution of one reaction by another without significant loss of the overall pathway productivity.

Robustness analysis:

COBRA Toolbox allows us to compute the effect of varying flux through a single reaction. The objective function changes as a flux through the particular reaction varies in magnitude, giving the idea of sensitivity and the overall importance of each reaction relative to the starch production[5],[6](Fig S4).

The resulting figure allows grouping of all the reactions into five classes according to their influence on the starch production rate: linear positive, linear negative, non-sensitive, one-side low rate saturation and one-side high rate saturation.

For each reaction of the linear starch biosynthetic pathway we can observe the same linear positive relationship with the starch production rate (Fig S4, reactions 6, 7, 8, 9, 10). This property emerges from the model and pathway structure (these reactions comprise the linear part of pathway and directly define the rate of starch biosynthesis). We determine these reactions to be extensively sensitive to small perturbations, and thus are attractive potential regulatory targets.

Reactions involved in branching demonstrate either positive (Fig S4, reactions 21, 25, 28, 29, 31, 33) or negative (Fig S4, reactions 17, 18, 23, 24, 27) saturation kinetics depending on whether they increase or decrease the starch production rate. Although both contribute to starch granule formation, the latter divert the precursors of the main starch components from the direct course. The starch synthesis rate is sustained near the optimum value for these reactions, indicating that the network is robust with respect to their flux changes. It is not surprising, for they comprise the networked structure of combinatorial events giving the same result: starch granule formation.

All the reactions participating in starch degradation give negative saturation behaviour as at a certain flux value they are responsible for diminution of the starch pool. As in the previous case, they describe the multichoice mechanism of complex glucan decomposition. As a result, the system as a whole is less sensitive to each of them. However, their importance for starch degradation correlates with the rate of the saturation. Some of them, such as reactions 41, 42, 47, 48, 50 (Alpha amylase and isoamylase) look to some degree accessible for regulation.

Finally, the reactions for beta-amylase and starch phosphorylase provide the sink of the system: the conversion of middle and short glucans to maltose and glucose. Therefore they demonstrate the highly sensitive, linear negative pattern and, similar to the synthetic reactions, appear to be a good target for regulation.

Sampling of solution space

We used the COBRA Toolbox for sampling of flux distributions that are allowed by stoichiometric properties of the network and given flux capacity constraints. The information obtained from the analysis is useful in respect of predicting the most likely flux value through the particular reaction, as well as the dependence relationships between reaction pairs. For

instance, CO_2 uptake is highly correlated with the reactions GAPphoto, which accounts for the photosynthesis, and GAP exchange, corresponding to the release of the half of the GAP to the cytosol (Fig S5, A). At the same time it is sufficiently independent from the reaction of GAP isomerisation, as well as the reaction for maltose production from 40LG glucans (40LGto maltose) is quite independent from reaction of maltose disproportionating (Fig S5).

Part 3

Evidence of the moderate redox regulation of starch pathway in O.tauri

When oxidised and dimerized the small subunit of AGPase is inactive, resulting in a lower affinity and decreased accessibility for allosteric regulation. In plants dimerization occurs through the disulfide bridge via Cys-82. The corresponding QTCL motif is well conserved in the N-terminal region of AGPB genes of almost all dicots and some monocots [12]. However, this motif is absent in *E.coli*, cyanobacteria and *Clamidomonas reinchardati*. [4, 5].Multiple alignment for the small AGPase subunit for the *Ostreococcus* and *Micromonas* species reveals that none of them contains the QTCL motif needed for Trx regulation (Fig 6). In their recent paper Kuhn at al, 2009 (Kuhn, 2009) provide experimental evidence that *O. tauri* ADP-glucose –phosphorylase is indeed not redox-regulated and speculate that redox regulation of this step appeared later in evolution.

Intriguingly, according to the sequence structures, this is also valid for starch degrading enzymes. Glucan, water dikinase (GWD), previously known as R1 protein is believed to play a key role in the process of starch mobilisation. GWD demonstrates reversible and specific Trx regulated binding to starch granules depending on the illumination state of the plant. The bound oxidized form is inactive and contains a disulfide bridge between C1004 and C1008. Chloroplast-located GWDs from potato, *Arabidopsis* and citrus share the sequence, that could not be detected in *Chlamydomonas* nor among the 5 GWD isoforms in *Ostreococcus* and *Micromonas* [13](Fig S13).

Another potential target for redox regulation is β-amylase, the starch degrading enzyme responsible for hydrolysis of soluble starch glycans by removal of maltose. The activity of one of the plastidic isoforms of *Arabidopsis* β-amylase, TR-BAMY, has been observed to be regulated by the cell redox state. The inactive form has a folded structure with the disulfide bridge between the Cys23 and Cys 470 [14]. Again, neither *Chlamydomonas*, nor *Ostreococcus* or *Micromonas* possess the analogues of this redox-sensitive isoform (Fig S14).

References

1. Ball SG, Morell MK: **FROM BACTERIAL GLYCOGEN TO STARCH: Understanding the Biogenesis of the Plant Starch Granule.** *Annual Review of Plant Biology* 2003, **54:**207-233.
2. Dumez S, Wattebled F, Dauvillee D, Delvalle D, Planchotb V, Ball SG, D'Hulst C: **Mutants of Arabidopsis Lacking Starch Branching Enzyme II Substitute Plastidial Starch Synthesis by Cytoplasmic Maltose Accumulation.** *The Plant Cell* 2006, **18:**2694-2709.
3. Ral J-P, Colleoni C, Wattebled F, Dauvillée D, Nempont C, Deschamps P, Li Z, Morell MK, Chibbar R, Purton S, et al: **Circadian Clock Regulation of Starch Metabolism Establishes GBSSI as a Major Contributor to Amylopectin Synthesis in Chlamydomonas reinhardtii.** *Plant Physiology* 2006, **142:**305-317.
4. Mikkelsen R, Mutenda KE, Mant A, Schürmann P, Blennow A: **α-Glucan, water dikinase (GWD): A plastidic enzyme with redox-regulated and coordinated catalytic activity and binding affinity.** *PNAS* 2005, **102:**1785-1790
5. Palsson BO: **Systems Biology: Properties of Reconstructed Networks** *Cambridge Univ Press* 2006.
6. Becker SA, Feist AM, Mo ML, Hannum G, Palsson BØ, Herrgard MJ: **Quantitative prediction of cellular metabolism with constraint-based models: the COBRA Toolbox.** *Nature Protocols* 2007, **2:**727 - 738
